# *Trichinella spiralis* cathepsin L induces macrophage M1 polarization via the NF-κB pathway and enhances the ADCC killing of newborn larvae

**DOI:** 10.1186/s13071-023-06051-1

**Published:** 2023-11-22

**Authors:** Ruo Dan Liu, Xiang Yu Meng, Chen Le Li, Qiu Yi Xu, Xin Zhi Lin, Bo Rang Dong, Chu Yan Ye, Tian Tian Miao, Xin Yi Si, Shao Rong Long, Jing Cui, Zhong Quan Wang

**Affiliations:** Department of Parasitology, Medical College, Zhengzhou University, Zhengzhou, 450052 China

**Keywords:** *Trichinella spiralis*, Cathepsin L, Macrophage polarization, NF-κB signaling pathway, ADCC

## Abstract

**Background:**

During the early stages of *Trichinella spiralis* infection, macrophages predominantly undergo polarization to the M1-like phenotype, causing the host’s inflammatory response and resistance against *T. spiralis* infection. As the disease progresses, the number of M2-type macrophages gradually increases, contributing to tissue repair processes within the host. While cysteine protease overexpression is typically associated with inflammation, the specific role of *T. spiralis* cathepsin L (TsCatL) in mediating macrophage polarization remains unknown. The aim of this study was to assess the killing effect of macrophage polarization mediated by recombinant *T. spiralis* cathepsin L domains (rTsCatL2) on newborn larvae (NBL).

**Methods:**

rTsCatL2 was expressed in *Escherichia coli* strain BL21. Polarization of the rTsCatL2-induced RAW264.7 cells was analyzed by enzyme-linked immunosorbent assay (ELISA), quantitative PCR (qPCR), western blot, immunofluorescence and flow cytometry. The effect of JSH-23, an inhibitor of nuclear factor kappa-light-chain-enhancer of activated B cells (NF-κB), on rTsCatL2-induced M1 polarization investigated. Cytotoxic effects of polarized macrophages on NBL were observed using in vitro killing assays.

**Results:**

Following the co-incubation of rTsCatL2 with RAW264.7 murine macrophage cells, qPCR and ELISA revealed increased transcription and secretion levels of inducible nitric oxide synthase (iNOS), interleukin (IL)-6, IL-1β and tumor necrosis factor alpha (TNF-α) in macrophages. Western blot analysis showed a significant increase in iNOS protein expression, while the expression level of arginase-1 protein remained unchanged. Flow cytometry revealed a substantial increase in the number of CD86-labeled macrophages. The western blot results also indicated that rTsCatL2 increased the expression levels of phospho-NF-κB and phospho-nuclear factor-κB inhibitor alpha (IκB-α) proteins in a dose-dependent manner, while immunofluorescence revealed that rTsCatL2 induced nuclear translocation of the p65 subunit of NF-κB (NF-κB p65) protein in macrophages. The inhibitory effect of JSH-23 suppressed and abrogated the effect of rTsCatL2 in promoting M1 macrophage polarization. rTsCatL2 mediated polarization of macrophages to the M1-like phenotype and enhanced macrophage adhesion and antibody-dependent cell-mediated cytotoxicity (ADCC) killing of NBL.

**Conclusions:**

The results indicated that rTsCatL2 induces macrophage M1 polarization via the NF-κB pathway and enhances the ADCC killing of NBL. This study provides a further understanding of the interaction mechanism between *T. spiralis* and the host.

**Graphical Abstract:**

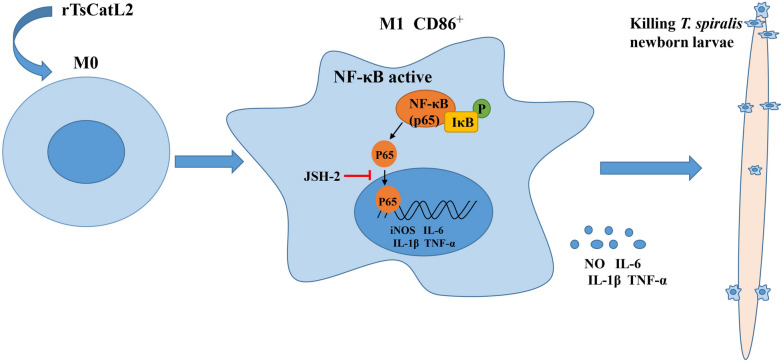

## Background

Trichinellosis is a severe zoonotic parasitic disease caused by eating raw or semi-raw animal meat infected with larvae of *Trichinella spiralis* [1]. Infected individuals usually suffer from abdominal pain, diarrhea, high fever, myalgia and allergic reactions, with potential complications that include pneumonia, encephalitis and myocarditis [2]. Trichinellosis is reported in over 55 countries worldwide, affecting over 11 million people [3]. Between 1986 and 2009, 65,818 cases of trichinellosis in humans were documented in 41 countries [4]. In China, trichinellosis is primarily prevalent in the southwest, northeast and central areas of the country, with eight outbreaks reported from 2009 to 2020, resulting in 479 infections and two deaths [5]. In Argentina, the number of suspected human cases reached 6662 during a trichinellosis outbreak between 2012 and 2018 [6]. In Bosnia and Herzegovina, 3828 people were reported to have been infected with trichinellosis from 1961 to 2021 [7]. In the Arctic territories of a Far Eastern District of the Russian Federation, a trichinellosis seroprevalence of 24.3% was detected in 259 people tested by an enzyme-linked immunosorbent assay (ELISA) [8]. Given this historical background, the public health impact of this disease poses a significant threat to human health and social stability, underscoring the importance of studying the proteins related to trichinellosis development.

*Trichinella spiralis* infection causes a cascade of immunological reactions in the host [9]. Macrophages, which are crucial innate immune cells and antigen-presenting cells, undergo polarization into classically activated macrophages (CAM or M1 phenotype) and alternatively activated macrophages (AAM or M2 phenotype) to fulfill distinct functions [10]. M1-type macrophages produce inflammatory cytokines that support an inflammatory response and promote parasite elimination, while M2-type macrophages dampen inflammation, aid in tissue repair and create conditions for parasite survival [11]. In the initial stages of trichinellosis, macrophages tend to polarize towards the M1-type phenotype, promoting an inflammatory response and disease resistance. As the infection progresses, the number of M2-type macrophages increases, contributing to tissue repair and participating in the parasite's immune evasion [12]. These distinctive macrophage polarization patterns substantially impact the host's immunological response and are crucial in predicting how infection will progress. Therefore, understanding host macrophage polarization is essential for effectively controlling trichinellosis and elucidating the interaction between *T. spiralis* and the host immune system.

Previous studies have indicated that cysteine cathepsin overexpression is often linked to inflammation, with cathepsin L in particular implicated in promoting inflammatory responses [13, 14]. Cathepsin L has been found to be involved in microglia-mediated neuroinflammation, and its inhibition effectively reduced the secretion of inflammatory cytokines such as inducible nitric oxide synthase (iNOS), interleukin (IL)-6 and tumor necrosis factor alpha (TNF-α) from microglia, leading to attenuated inflammation [14]. *Fasciola hepatica* cathepsin L3 has been demonstrated to mediate the activation of dendritic cell inflammasomes and the production of IL-1β and IL-18 [15], and *Giardia duodenalis* cathepsin L was shown to induce the release of nitric oxide (NO) and pro-inflammatory factors from the J774A.1 murine macrophage cell line [16]. Moreover, *T. spiralis* cystatin (Ts-Cys) activates M2 macrophages through suppressing the TLR2/MyD88 signal pathway [17]. *Trichinella spiralis* cystatin (TsCstN) inhibited the production of pro-inflammatory cytokines and the expression of MHC class II molecules in mouse bone marrow-derived macrophages [18]. Notably, *T. spiralis* cathepsin L (TsCatL) has been identified as a crucial digestive enzyme that breaks down hemoglobin serum albumin, immunoglobulin and extracellular matrix components in vitro [19, 20]. However, the specific role of TsCatL in regulating macrophage polarization in the host is still unclear.

In the present study we investigated the effect of recombinant domains of TsCatL (rTsCatL) on polarizing macrophages and activating the nuclear factor kappa-light-chain-enhancer of activated B cells (NF-κB) pathway. In vitro, research was done to determine if rTsCatL2-induced M1-type macrophage polarization resulted in the death of *T. spiralis* newborn larvae (NBL). This study offers a theoretical framework for preventing and controlling trichinellosis and enhances our understanding of host-parasite interaction.

## Methods

### Parasites, cells and animals

Swine-derived *T. spiralis* isolate T1 (ISS534) was passaged in Kunming mice bred in our laboratory [21]. Mouse monocyte-macrophage leukemia cells (RAW264.7 murine macrophage cell line) were obtained from Procell Life Science & Technology Co., Ltd (Wuhan, China). Female Kunming mice aged 4–6 weeks were procured from the Huaxing Animal Center.

### Expression and characterization of rTsCatL2 protein

Recombinant bacteria BL21-pQE-80L/TsCatL2 (*E. coli* strain BL21 induced using the pQE-80L expression vector system/TsCatL2) were induced at 22 °C with the addition of 0.1 mM IPTG, with shaking at 100 rpm. After induction, the bacteria were collected, and the rTsCatL2 was purified by Ni-NTA Beads 6FF (Solarbio, Beijing, China) [22]. The isolated rTsCatL2 protein was evaluated using sodium dodecyl sulfate-polyacrylamide gel electrophoresis (SDS-PAGE) and western blot. Nitrocellulose filter (NC) membranes containing rTsCatL2 were incubated with primary antibodies overnight. Mouse anti-rTsACatL2 serum was prepared by immunizing BALB/c mice with rTsCatL2 mixed with adjuvant by intramuscular injection and then collecting the blood of the mice to separate out the serum. The monoclonal antibodies were purchased from Servicebio [20]. The membranes were then treated with horse radish peroxidase (HRP)-anti-mouse immunoglobulin G (IgG) (Proteintech, Rosemont, IL, USA), followed by staining with DAB (Solarbio) [23].

### Mass spectrometry detection

The purified rTsCatL2 protein was assessed using SDS-PAGE. After electrophoresis, corresponding bands of rTsCatL2 on the colloid were excised and the surrounding blank strips were removed as much as possible. The strips were placed in a centrifuge tube, sealed and sent to Sangon Biotech (Shanghai, China) for detection by liquid chromatography-tandem mass spectrometry (LC-MS/MS) [24].

### Cell culture

RAW264.7 cells were cultured in DMEM medium supplemented with 10% fetal bovine serum (Biological Industries, Kibbutz Beit-Haemek, Israel) [25]. M1-type macrophages were obtained by co-culturing the RAW264.7 cells with 100 ng/ml lipopolysaccharide (LPS; Solarbio, China) + 20 ng/ml interferon gamma (IFN-γ; BioLegend, San Diego, CA, USA), and M2-type macrophages were obtained following co-culture with 40 ng/ml IL-4 (BioLegend, USA) [26].

### Cell viability test

Endotoxin removal resin (Thermo Fisher Scientific, Waltham, MA, USA) was used to eliminate endotoxin interference in rTsCatL2. The endotoxin level in rTsCatL2 was measured using the Limulus Amebocyte Lysate Kit (Bioendo Technologies, Xiamen, China), and the results indicated an endotoxin level of < 0.05 EU/ml, suggesting a minimal influence on macrophages [27]. RAW264.7 cell viability was assessed using the Cell Counting Kit-8 (CCK-8; TargetMol, Boston, MA, USA) [28]. RAW264.7 cells were cultured in 96-well plates and incubated with LPS (100 ng/ml) and different concentrations of rTsCatL2 (1–10 μg/ml) for various time periods (12, 24, 36, and 48 h). Cell viability was determined using readings from a multifunctional enzyme-labeled instrument (Tecan, Zurich, Switzerland) at 450 nm.

### Cell immunofluorescence assay of rTsCatL2 and p65 nuclear translocation

RAW264.7 macrophages were grown on glass coverslips and co-cultured with 5 μg/ml rTsCatL2 or phosphate-buffered saline (PBS) for 1 h, following which the cells were fixed with 4% paraformaldehyde. Antigen repair buffer (100 mM Tris, 5% urea, pH 9.5) was added, and the cells were microwaved at 95 °C for 10 min. The cells were then treated with a PBS solution containing 0.2% Triton X-100 for 10 min [29]. Blocking solution (1% bovine serum albumin [BSA] and 22.52 mg/ml glycine in PBST) was added to the cells for 30 min, followed by the addition of mouse anti-rTsCatL2 immune serum for 1 h. The cells were cleaned with PBS before being treated with FITC-anti-mouse IgG [30, 31]. Nuclei were labeled with propidium iodide (PI; Solarbio) for 1 min. The internalization of rTsCatL2 in RAW264.7 macrophages was visible under a fluorescent microscope.

For translocation of nuclear transcription factor p65 (NF-κB p65), RAW264.7 cells were treated with 5 μg/ml rTsCatL2 for various durations (0.5, 1 and 6 h) or with different concentrations of rTsCatL2 (0.5, 1, 3 and 5 μg/ml) for 1 h. Negative control (PBS) and positive control (LPS) cells were also included. Anti-NF-B p65 (Abcam, Cambridge, UK) was used as the main antibody in an immunofluorescence experiment, and FITC-conjugated goat anti-rabbit IgG (Proteintech) was used as the secondary antibody. After PI staining, the effect was observed under a fluorescence microscope.

### Western blotting of iNOS, arginase 1 proteins and signaling pathway

RAW264.7 cells were harvested after being subjected to various stimuli, and cellular proteins were extracted using RAPI lysate (Beyotime, Haimen, Jiangsu, China). The protein concentration was determined using the BCA Assay Kit (Beyotime), and 20-g samples of proteins were prepared for SDS-PAGE and western blot analysis [32]. After blocking with blocking buffer (Epizyme, Shanghai, China), the PVDF membranes were incubated with primary antibodies against iNOS (Abcam), arginase 1 (Arg-1; Hua Bio, Hangzhou, China), NF-κB p65, phospho-NF-κB p65 (pNF-κB p65), nuclear factor-κB inhibitor alpha (IκB-α), phospho-IκB-α (p-IκB-α; Abcam) and β-actin antibody (Servicebio, Wuhan, China) [33]. Following incubation with HRP-conjugated goat anti-mouse/rabbit IgG (Proteintech), the PVDF membranes were exposed. Membranes were scanned with an automated chemiluminescence image analyzer and quantified using ImageJ software [34].

### NO quantification

Nitric oxide secretion by RAW264.7 cells was determined using a NO assay kit (Beyotime). Cell culture supernatants from different treatment groups were collected, and a 50-μl aliquot of NaNO_2_ standard (range: 1–100 μM) or supernatant was added to each well of a 96-well plate. Griess Reagents I and II were then added to each well, and absorbance was detected at 540 nm. The standard curve was constructed and NO concentration in the samples was calculated.

### Quantitative PCR

RNA was extracted by TRNzol (TIANGEN Biotech (Beijing) Co., Ltd., Beijing, China), followed by reverse transcription into complementary DNA (cDNA) with the PrimeScript™ RT kit (Takara Bio Inc., Shiga, Japan) [23]. Quantitative PCR (qPCR) primers for IL-1β, IL-6, iNOS, TNF-α, Arg-1, transforming growth factor beta (TGF-β) and mouse glyceraldehyde 3-phosphate dehydrogenase (GAPDH) were synthesized by Shanghai Biotech (Table 1). The qPCR were carried out with TB Green® Premix Ex Taq™ (Takara Bio Inc.) on the 7500 Real-Time PCR Systems (Applied Biosystems, Thermo Fisher Scientific, Waltham, MA, USA). Relative transcript levels of genes from different treatment groups were calculated using the 2^−ΔΔCt^ method [35].

**Table 1:** Primer sequences for the quantitative PCR

Prime name	Sequence (5′–3′)
iNOS	F: GAGACAGGGAAGTCTGAAGCAC R: CCAGCAGTAGTTGCTCCTCTTC
IL-1 β	F: TGGACCTTCCAGGATGAGGACA R: GTTCATCTCGGAGCCTGTAGTG
IL-6	F: TACCACTTCACAAGTCGGAGGC R: CTGCAAGTGCATCATCGTTGTTC
TNF-α	F: TCTTCTCATTCCTGCTTGTGG R: CACTTGGTGGTTTGCTACGA
Arg-1	F: CATTGGCTTGCGAGACGTAGAC R: GCTGAAGGTCTCTTCCATCACC
TGF-β	F: TGATACGCCTGAGTGGCTGTCT R: CACAAGAGCAGTGAGCGCTGAA
GAPDH	F: GGTTGTCTCCTGCGACTTCA R: TGGTCCAGGGTTTCTTACTCC

*ARG-1* Arginase 1,* F* forward,* GAPDH *glyceraldehyde 3-phosphate dehydrogenase,* iNOS* inducible nitric oxide synthase,* IL* interleukin, *R* reverse, *TGF-β* transforming growth factor beta,* TNF-α* tumor necrosis factor alpha

### Enzyme-linked immunosorbent assay

Cell culture supernatants from different experimental groups were collected, and the levels of IL-6, IL-1β, TNF-α, IL-10 and TGF-β secreted were quantified using mouse cytokine ELISA kits (Proteintech). The microplate reader (Tecan, Switzerland, China) was calibrated at a wavelength of 630 nm, and the optical density (OD) was measured at 450 nm. ELISACalc software was used to fit the standard concentration as the horizontal coordinate against the zeroed OD as the vertical coordinate, facilitating the calculation of sample concentrations based on their respective OD values [36].

### Flow cytometry

RAW264.7 macrophages (1 × 10^6^) were transferred to a 6-well plate and incubated for 24 h, following which several reagents were added and stimulation lasted for 24 h. The experimental groups were treated with rTsCatL2 (5 ng/ml), LPS (100 ng/ml) + IFN-γ (20 ng/ml), IL-4 (40 ng/ml) and PBS (control), respectively. Cells from different experimental groups were washed with FACS buffer containing PBS with 0.1% BSA and 0.5 mM EDTA. Purified rat anti-mouse CD16/CD32 blocking agent (BD Biosciences, Franklin Lakes, NJ, USA) was then added and the macrophages incubated for 10 min. APC anti-mouse F4/80 (BioLegend) and PE anti-mouse CD86 (BioLegend) flow cytometry antibodies were then added, and the macrophages were incubated on ice for 20 min. After incubation, the cells were permeabilized using the FIX & PERM Kit (Lianke Bio, China). FITC anti-mouse CD206 antibody (BioLegend) was then added sequentially and the cells incubated on ice at 4 °C for 20 min in the dark, following which FACS buffer was added to resuspend the cells. The samples were analyzed using the BD FACSCanto™ II Flow Cytometer system (BD Biosciences) and FlowJo software [37].

### Inhibition of rTsCatL2-mediated RAW264.7 M1 polarization by JSH-23

RAW264.7 macrophages were first cultured in 6-well plates for 24 h and then incubated with 30 μM JSH-23, an inhibitor of NF-κB, for 6 h; 5 μg/ml rTsCatL2 was then added to evaluate the inhibitory effect of JSH-23 on the NF-κB pathway. Separate treatment groups, consisting of PBS, JSH-23, LPS and rTsCatL2, respectively, were included as controls [38]. After incubating the cells for 1 h with the different reagents, the phophorylation levels of NF-κB protein was determined by western blot. Additionally, the effect of expression of the macrophage surface marker CD86 was determined by flow cytometry after 24 h of incubation with each reagent, and the impact on macrophage transcription levels of pro-inflammatory factors iNOS, IL-6, IL-1β, and TNF-α was determined by qPCR. The Griess method and ELISA were used to determine the levels of NO, IL-6, IL-1β and TNF-α secreted by macrophages. Differences in iNOS expression were examined by western blot.

### Killing effect of macrophages on NBL

RAW264.7 cells were separately exposed to PBS, IL-4, LPS + IFN-γ and rTsCatL2 for 6 h. In each group, 1 × 10^5^ macrophages and 50 NBL were mixed and added to 48-well plates, followed by incubation for 96 h. Macrophage adhesion and the survival status of NBL were determined by microscopy. Surviving NBL exhibited an active and wriggling behavior, while dead NBL remained erect and immobile [39]. Then, the synergistic killing effect of macrophages and complement was investigated using fresh guinea pig serum as normal serum (S). Inactivated complement sera (H.S) were obtained by heating normal sera in a water bath at 56 °C for 30 min. Macrophages from the different treatment groups were added to S (1:100) or H.S (1:100) to observe macrophage adhesion and NBL killing. Lastly, the role of antibody-dependent cell-mediated cytotoxicity (ADCC) involving macrophages was explored. Macrophages in the different treatment groups were incubated with rTsCatL2 immune serum (I.S) (1:100) and 50 NBL for 96 h, followed by microscopic observation [40]. Cytotoxicity was determined by dividing the proportion of dead NBL by the total number of worms in each assay, which was carried out in triplicate. Each experiment was performed in triplicate.

### Statistical analysis

Experimental data were graphed using GraphPad Prism 8.0 software (GraphPad Software, San Diego, CA, USA) and statistical analysis was performed using SPSS 21.0 software (SPSS IBM Corp., Armonk, NY, USA). The one-way analysis of variance (ANOVA), independent samples t-test, linear regression and Chi-squared test (*χ*^2^) were among the statistical analysis techniques employed in the study. The threshold for statistical significance was set at *P* < 0.05.

## Results

### Expression and identification of rTsCatL2

**Fig. 1:**
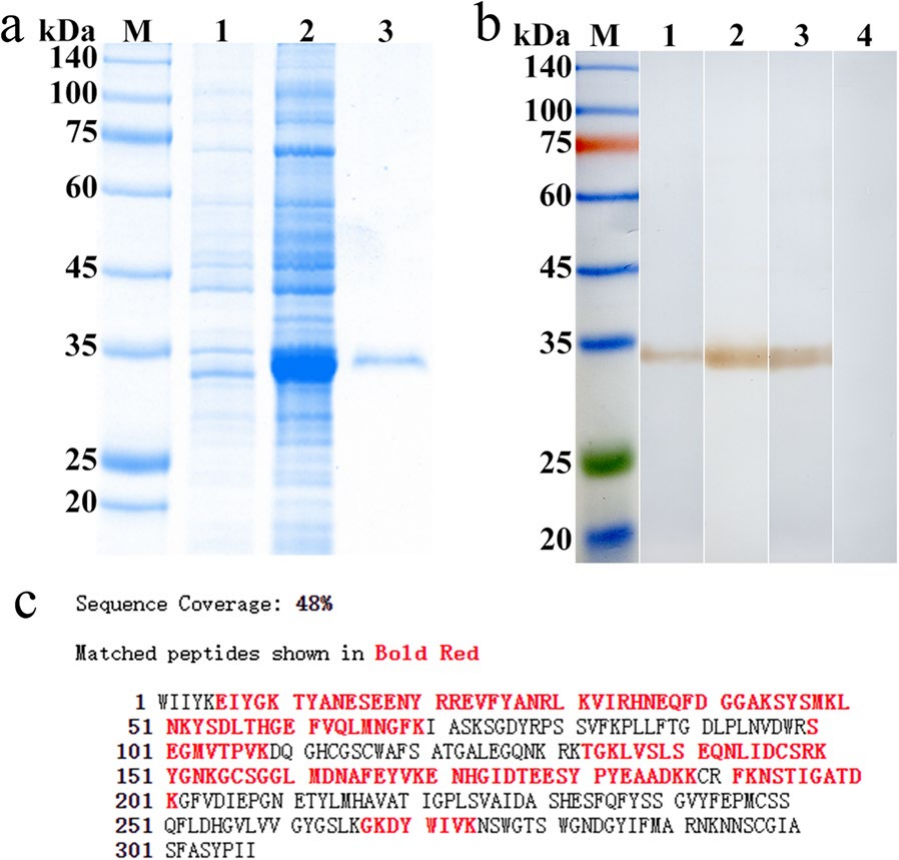
Expression and identification of rTsCatL2. **a** Results of SDS-PAGE. Lanes: M, marker; 1, recombinant bacterial pQE-80L/TsCatL2 lysate before induction; 2, pQE-80L/TsCatL2 lysate after 0.1 mM IPTG induction at 22 °C; 3, purified rTsCatL2. **b** Results of western blot. Lanes: M, Marker; 1, infection with *Trichinella spiralis* serum; 2, anti-rTsCatL2 serum; 3, His monoclonal antibody; 4, normal mouse serum. **c** Detection of rTsCatL2 by LC–MS/MS. Mass spectrometry results for rTsCatL2 were compared with the amino acid sequence of the cathepsin L (KRY31298.1) in the Mascot search engine (V2.3.02). The matched peptide is shown in red, and its sequence coverage reached 48%. LC–MS/MS, Liquid chromatography-tandem mass spectrometry; rTsCatL2, Recombinant *T. spiralis* cathepsin L domains; SDS-PAGE, sodium dodecyl sulfate-polyacrylamide gel electrophoresis

The expression of rTsCatL2 was induced and the protein purified using a Ni column. SDS-PAGE confirmed the purification, showing a single protein band at approximately 34 kDa (Fig. 1a). Western blot analysis demonstrated that rTsCatL2 specifically reacted with *T. spiralis* infection serum, rTsCatL2 immune serum and His monoclonal antibody, but not with normal serum (Fig. 1b). The amino acid sequence detected by LC–MS/MS was then inputted into the Mascot (V2.3.02) search engine for protein identification, resulting in a match with 56 proteins, with *T. spiralis* cathepsin L (KRY31298.1) obtaining the highest score.

### Cell viability of RAW264.7

**Fig. 2:**
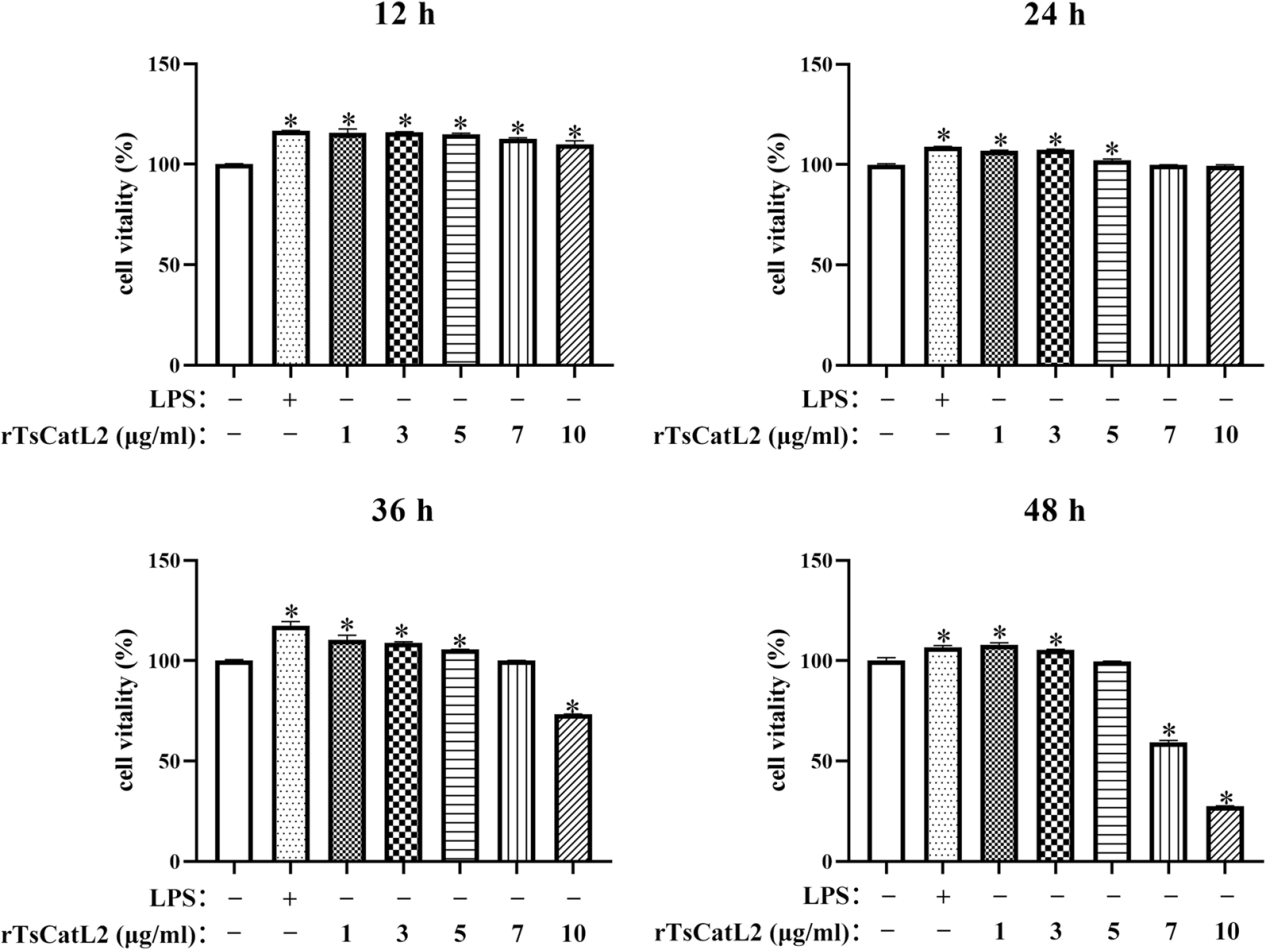
Effect of different concentrations of rTsCatL2 on the viability of RAW264.7 macrophages. The viability of RAW264.7 cells in different experimental groups after treatment with rTsCatL2 for 12, 24, 36 and 48 h, respectively. Asterisks indicates statistically significant difference at * *P* < 0.001 compared to the blank control group. LPS, lipopolysaccharide; RAW264.7, murine macrophage cell line

The results of the CCK-8 assay showed that incubating RAW264.7 cells with 1–5 μg/ml rTsCatL2 for 36 h significantly increased cell viability compared to the control cells (ANOVA; *F*_12h_ = 92.622, *F*_24h_ = 284.053, *F*_36h_ = 388.102; *P* < 0.001) (Fig. 2). When rTsCatL2 at a concentration of 5 μg/ml was co-incubated with RAW264.7 cells for 48 h, there was no significant decrease in cell viability compared to the control group (*P* > 0.05). However, when the concentration of rTsCatL2 was ≥ 7 μg/ml, cell viability was significantly lower than that of the control group (ANOVA; *F* = 3494.253; *P* < 0.001) (Fig. 2). Consequently, the working concentration of rTsCatL2 for the RAW264.7 cell experiment was set at 5 μg/ml.

### RAW264.7 internalizes rTsCatL2

**Fig. 3:**
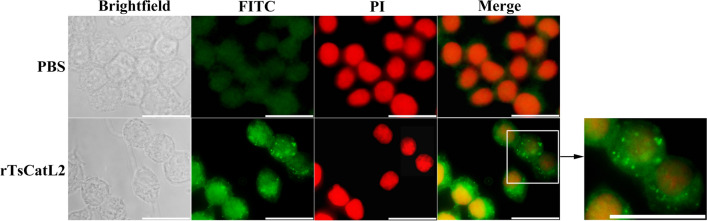
Immunofluorescence analysis of rTsCatL2 internalization in RAW264.7 macrophages. RAW264.7 macrophages were incubated with rTsCatL2 (5 μg/ml) for 1 h; the resulting green fluorescence showed that rTsCatL2 was internalized by RAW264.7 macrophages. Scale bar: 20 μm. FITC, Fluorescein isothiocyanate; PBS, phosphate-buffered saline; PI, propidium iodide; RAW264.7, murine macrophage cell line; rTsCatL2, recombinant *T. spiralis* cathepsin L domains

The cellular immunofluorescence study revealed a bright green fluorescence in the cells treated with rTsCatL2, indicating internalization of this protein by macrophages (Fig. 3).

### rTsCatL2 increases the level of M1 cytokine transcription

**Fig. 4:**
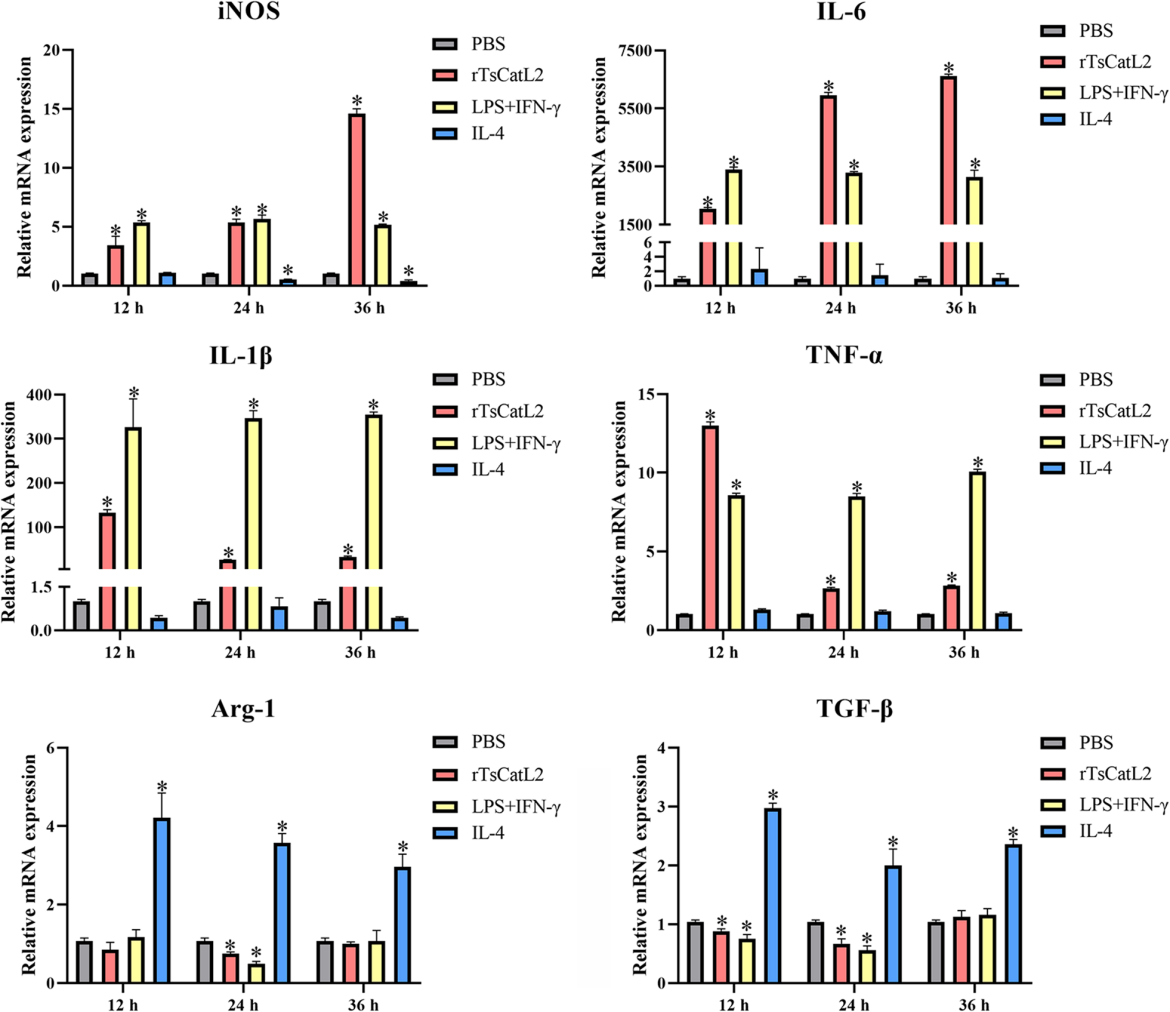
qPCR analysis of transcriptional levels of cytokines of RAW264.7 macrophages incubated with rTsCatL2. The transcription levels of iNOS, IL-6, IL-1β, TNF-α, Arg-1 and TGF-β were detected in different groups of RAW264.7 cells. * *P* < 0.05 compared with the PBS group. Arg-1, Arginase-1; iNOS, inducible nitric oxide synthase; IL, interleukin; LPS, lipopolysaccharide; PBS, phosphate-buffered saline; RAW264.7, murine macrophage cell line; rTsCatL2, recombinant *T. spiralis* cathepsin L domains; TGF-β, transforming growth factor beta; TNF-α, tumor necrosis factor alpha

The qPCR analysis demonstrated that the stimulation of RAW264.7 cells with rTsCatL2 led to an increase in transcript levels of the iNOS, IL-6, IL-1β and TNF-α genes (Fig. 4). Specifically, after 12, 24 and 36 h of rTsCatL2 stimulation, the iNOS gene transcription levels increased by 3.5-, 5.2- and 14.0-fold, respectively (ANOVA; *F*_12h_ = 86.458, *F*_24h_ = 543.930, *F*_36h_ = 2714.131; *P* < 0.001). Similarly, the messenger RNA (mRNA) transcript levels of IL-6 increased by 2060-, 5975- and 6645-fold after 12, 24 and 36 h of rTsCatL2 stimulation, respectively. Additionally, the mRNA transcript levels of IL-1β increased by 133-, 26- and 32-fold after 12, 24 and 36 h of rTsCatL2 stimulation, respectively (ANOVA; *F*_12h_ = 69.207, *F*_24h_ = 1195.156, *F*_36h_ = 9682.555; *P* < 0.05). The mRNA transcript levels of TNF-α increased by 12.7-, 2.6- and 2.8-fold after 12, 24 and 36 h of rTsCatL2 stimulation, respectively. (ANOVA; *F*_12h_ = 5839.247, *F*_24h_ = 3161.714, *F*_36h_ = 6761.298; *P* < 0.001). Furthermore, rTsCatL2 stimulation resulted in a decrease in Arg-1 gene transcription at 24 h (ANOVA; *F* = 348.906; *P* < 0.05) and a reduction in the transcription level of the TGF-β gene at 12 and 24 h (ANOVA; *F*_12h_ = 906.067, *F*_24h_ = 56.395; *P* < 0.05). These findings collectively indicate that rTsCatL2 promotes the transcription of M1-type macrophage factor genes in RAW264.7 cells.

### rTsCatL2 promotes iNOS protein expression

**Fig. 5:**
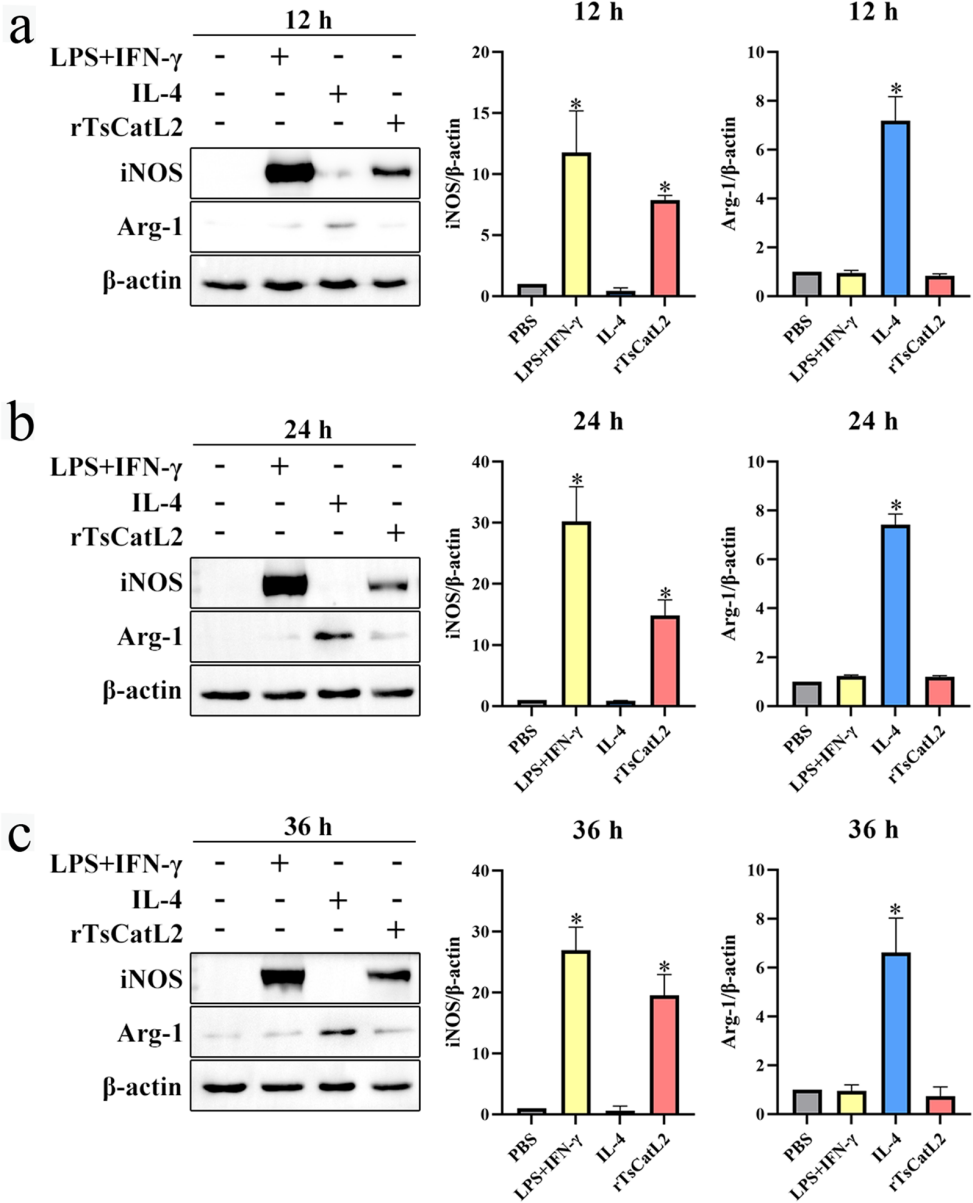
Western blotting of expression levels of iNOS and Arg-1 in RAW264.7 macrophages incubated with rTsCatL2. The expression levels of iNOS and Arg-1 protein in RAW264.7 cells were detected after 12 h (**a**), 24 h (**b**) and 36 h (**c**) of treatment in the different groups. The asterisk indicates a statistically significant difference at *P < 0.05 compared to the PBS group (control). Arg-1, Arginase-1; iNOS, inducible nitric oxide synthase; INF, interferon; IL, interleukin; LPS, lipopolysaccharide; PBS, phosphate-buffered saline; RAW264.7, murine macrophage cell line; rTsCatL2, recombinant *T. spiralis* cathepsin L domains

The results of the western blot analysis demonstrated a significant increase in iNOS protein expression in both the LPS + IFN-γ group and the rTsCatL2 group (Fig. 5). Following 12, 24 and 36 h of rTsCatL2 stimulation, iNOS protein expression was 7.9-, 14.8- and 20.0-fold higher than that of the PBS group (control), respectively (ANOVA; *F*_12h_ = 87.815, *F*_24h_ = 60.232, *F*_36h_ = 79.583; *P* < 0.01). There was no significant difference in iNOS protein expression between the IL-4 and PBS groups (*P* > 0.05). In addition, the expression level of Arg-1 protein increased in the IL-4 group compared to the PBS group (ANOVA; *F*_12h_ = 119.164, *F*_24h_ = 575.914; *F*_36h_ = 45.005; *P* < 0.01). However, there was no significant difference in Arg-1 protein expression between the rTsCatL2 and PBS groups (*P* > 0.05). These findings demonstrate that rTsCatL2 can promote the expression of iNOS protein of the M1 marker in RAW264.7 cells.

### rTsCatL2 increases the level of M1 cytokine secretion

**Fig. 6:**
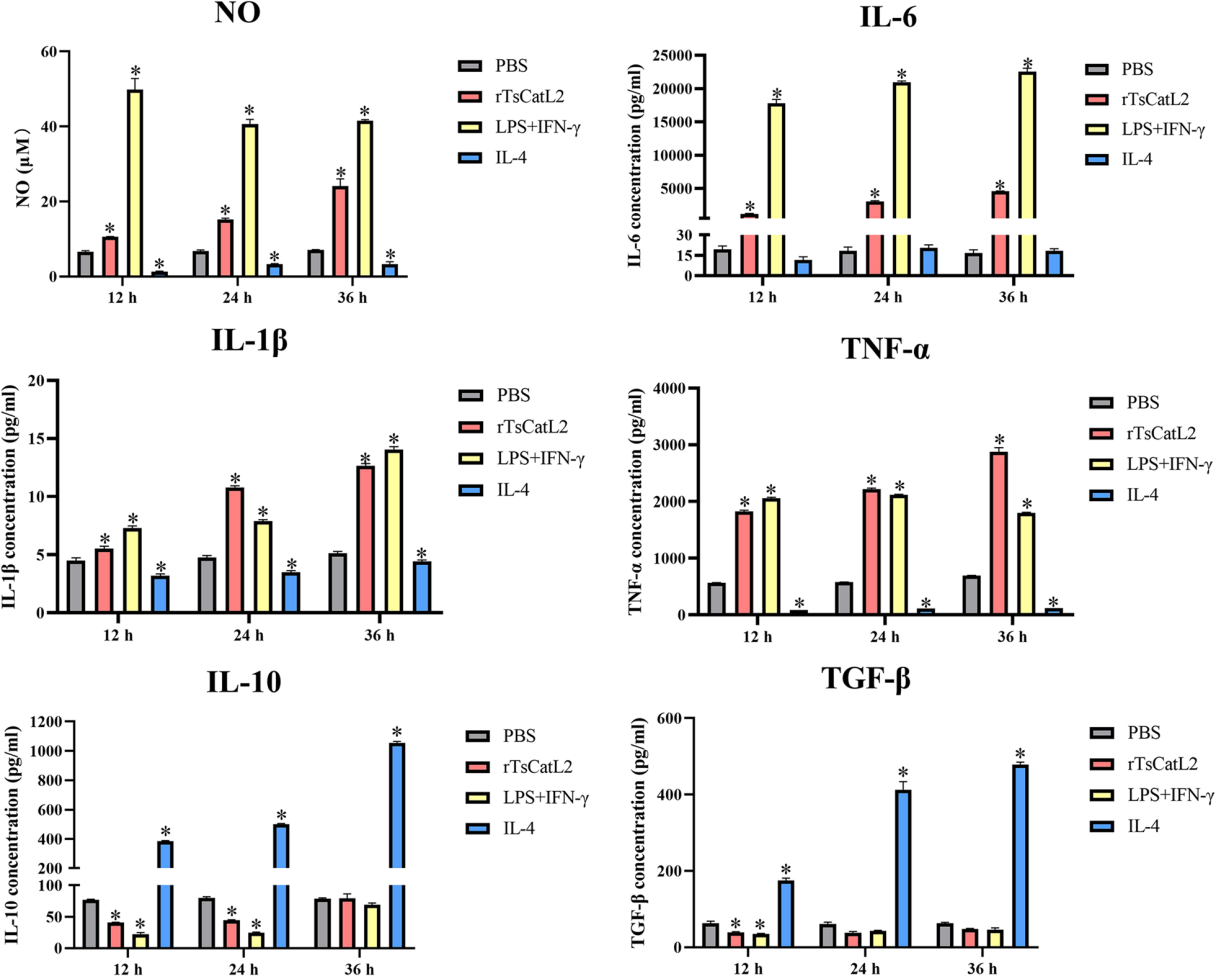
Effects of rTsCatL2 on the secretion of cytokines and NO by RAW264.7 macrophages. The secretion levels of NO, IL-6, IL-1β, TNF-α, IL-10 and TGF-β in different experimental groups of RAW264.7 cells are shown. This asterisk indicates a statistically significant difference at *P < 0.05 compared to the PBS group. NO, Nitric oxide; INF, interferon; IL, interleukin; LPS, lipopolysaccharide; PBS, phosphate-buffered saline; RAW264.7, murine macrophage cell line; rTsCatL2, recombinant *T. spiralis* cathepsin L domains; TGF-β, transforming growth factor beta; TNF-α, tumor necrosis factor alpha

The Griess and ELISA methods were used to determine rTsCatL2-mediated cytokine expression in RAW264.7 cells (Fig. 6). At 12, 24 and 36 h after rTsCatL2 co-incubation of cells, the levels of NO secretion reached 10.6, 15.2 and 24.1 μM, respectively, which were 1.6-, 2.2- and 3.4-fold higher than those in the PBS group (ANOVA; *F*_12h_ = 658.598, *F*_24h_ = 1902.432, *F*_36h_ = 866.814; *P* < 0.001). The levels of IL-6 cytokine secretion in the rTsCatL2 group were 1189.2, 3204.4, and 4575.5 μM, respectively, indicating a 60.7-, 163.4- and 274.7-fold increase over those in the PBS group (ANOVA; *F*_12h_ = 2645.648, *F*_24h_ = 22,294.644, *F*_36h_ = 5893.901; *P* < 0.01). Regarding IL-1β cytokine secretion, the levels in the rTsCatL2 group were 5.4, 10.8 and 12.6 μM, respectively, reflecting increases of 1.2-, 2.3- and 2.5-fold over those in the PBS group (ANOVA; *F*_12h_ = 231.284,* F*_24h_ = 1661.714,* F*_36h_ = 2226.356; *P* < 0.01). Moreover, the TNF-α cytokine secretion levels in the rTsCatL2 group were 1828.0, 2216.9 and 2874.7 μM, respectively, which were 3.2-, 3.8- and 4.1-fold higher than those in the PBS group (ANOVA; *F*_12h_ = 13,994.073, *F*_24h_ = 25,779.706, *F*_36h_ = 3168.54;* P* < 0.001). Additionally, rTsCatL2 inhibited IL-10 secretion after 12 and 24 h of stimulation (ANOVA; *F*_12h_ = 13,131.29, *F*_24h_ = 37,595.833; *P* < 0.001) and also suppressed TGF-β secretion after 12 h of stimulation (ANOVA; *F*_12h_ = 632.685; *P* < 0.01). The secretion levels of IL-6, IL-1β, TNF-α and NO were increased in the LPS + IFN-γ group (*P* < 0.05), and those of IL-10 and TGF-β were increased in the IL-4 group (*P* < 0.001).

### rTsCatL2 promotes the proliferation of CD86-labeled macrophages

**Fig. 7:**
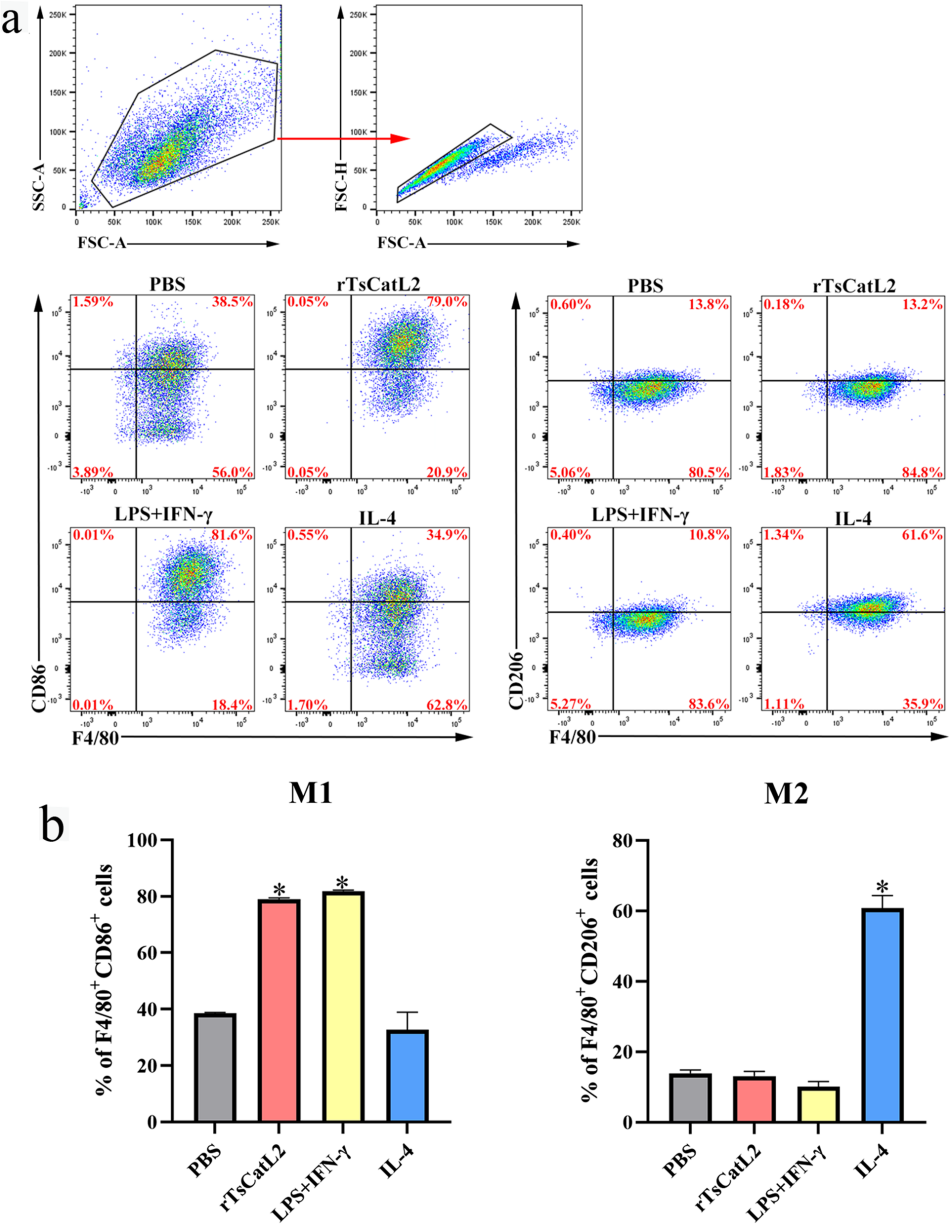
Flow cytometric analysis of changes in M1- and M2-related surface molecules in RAW264.7 macrophages incubated with rTsCatL2. **a** FlowJo software version 10.8.1 was used to analyze the macrophage population in the different treatment groups. **b** Percentage of M1 and M2 macrophages in the different treatment groups. The asterisk indicates a statistically significant difference at *P < 0.05 compared with the PBS group. INF, interferon; IL, interleukin; LPS, lipopolysaccharide; PBS, phosphate-buffered saline; RAW264.7, murine macrophage cell line; rTsCatL2, recombinant *T. spiralis* cathepsin L domains

Flow cytometry analysis revealed an increase in CD86-labeled macrophages in both the LPS + IFN-γ and rTsCatL2 groups compared to the PBS group (ANOVA; *F* = 204.995; *P* < 0.001). The number of CD206-labeled macrophages also significantly increased in the IL-4 group (ANOVA; *F* = 409.799, *P* < 0.001) (Fig. 7). However, there was no noticeable change in the number of CD206-labeled macrophages after rTsCatL2 stimulation when compared to the PBS group (*P* > 0.05). These findings further support that rTsCatL2 stimulates RAW264.7 cells toward M1-type polarization.

### rTsCatL2 promotes protein phosphorylation in the NF-κB signaling pathway

**Fig. 8:**
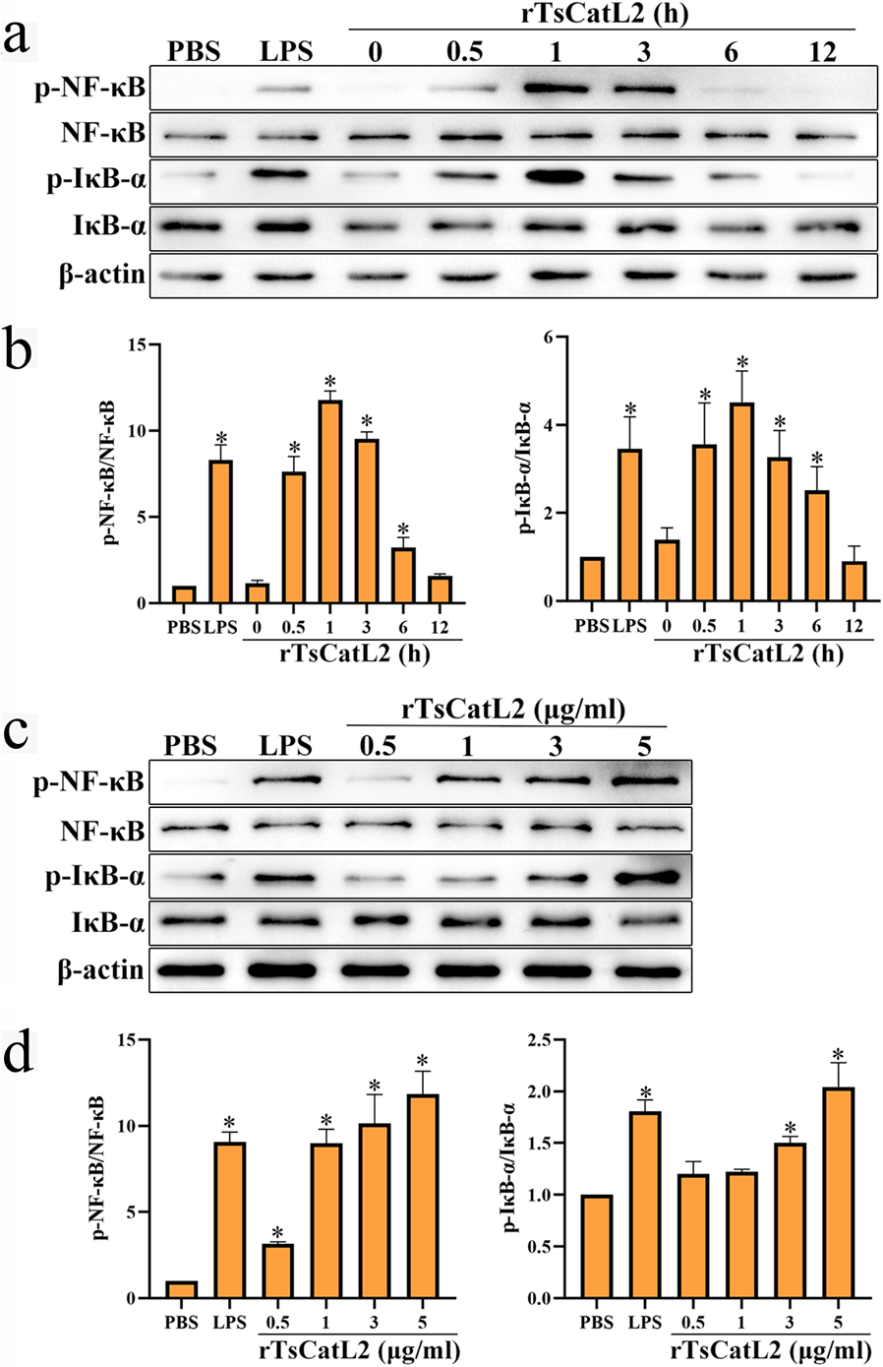
Western blot analysis of NF-κB pathway activation in RAW264.7 macrophages incubated with rTsCatL2.** a**,** b** Expression levels of p-NF-κB and p-IκB-α in RAW264.7 macrophages incubated with rTsCatL2 for 0—12 h.** c**,** d** Expression levels of p-NF-κB and p-IκB-α in RAW264.7 macrophages incubated with different concentrations of rTsCatL2 (range: 0.5–5 μg/ml). The asterisk indicates a statistically significant difference at *P < 0.05 compared with the PBS group. IκB-α, Nuclear factor-κB inhibitor alpha; LPS, lipopolysaccharide; NF-κB, nuclear factor kappa-light-chain-enhancer of activated B cells; p, phorphorylated; PBS, phosphate-buffered saline; RAW264.7, murine macrophage cell line; rTsCatL2, recombinant *T. spiralis* cathepsin L domains

RAW264.7 cells were treated with rTsCatL2 for 0, 0.5, 1, 3, 6 and 12 h, respectively, to examine the dynamic activation of the NF-κB pathway. The phosphorylation of NF-κB and IκB-α by rTsCatL2 commenced at 0.5 h after treatment initiation and lasted for 6 h. Peak phosphorylation of NF-κB and IκB-α was reached at 1 h, with 11.77- and 4.43-fold increase compared to the PBS group, respectively (ANOVA; *F*_NF-κB_ = 181.713, *F*_IκB-α_ = 15.612; *P* < 0.001). However, at 12 h, phosphorylation of NF-κB and IκB-α in the rTsCatL2 group did not significantly differ from that in the PBS group (*P* > 0.05) (Fig. 8a, b). We then examined the impact of different concentrations of rTsCatL2 (0.5, 1, 3 and 5 μg/ml) on the activation of the NF-κB signaling pathway. rTsCatL2 was found to enhance the phosphorylation levels of NF-κB and IκB-α proteins in a dose-dependent manner (*r*_NF-κB_ = 0.793, *r*_IκB-α_ = 0.93; *P* < 0.01). The highest phosphorylation levels of NF-κB and IκB-α were reached following stimulation with 5 μg/ml rTsCatL2, increasing to 10.09- and 2.03-fold that of the PBS group, respectively (ANOVA; *F*_NF-κB_ = 58.957, *F*_IκB-α_ = 33.078; *P* < 0.05) (Fig. 8c, d). These results indicate that rTsCatL2 promotes protein phosphorylation in the NF-κB signaling pathway.

### rTsCatL2 promotes nuclear translocation of p65 protein

**Fig. 9:**
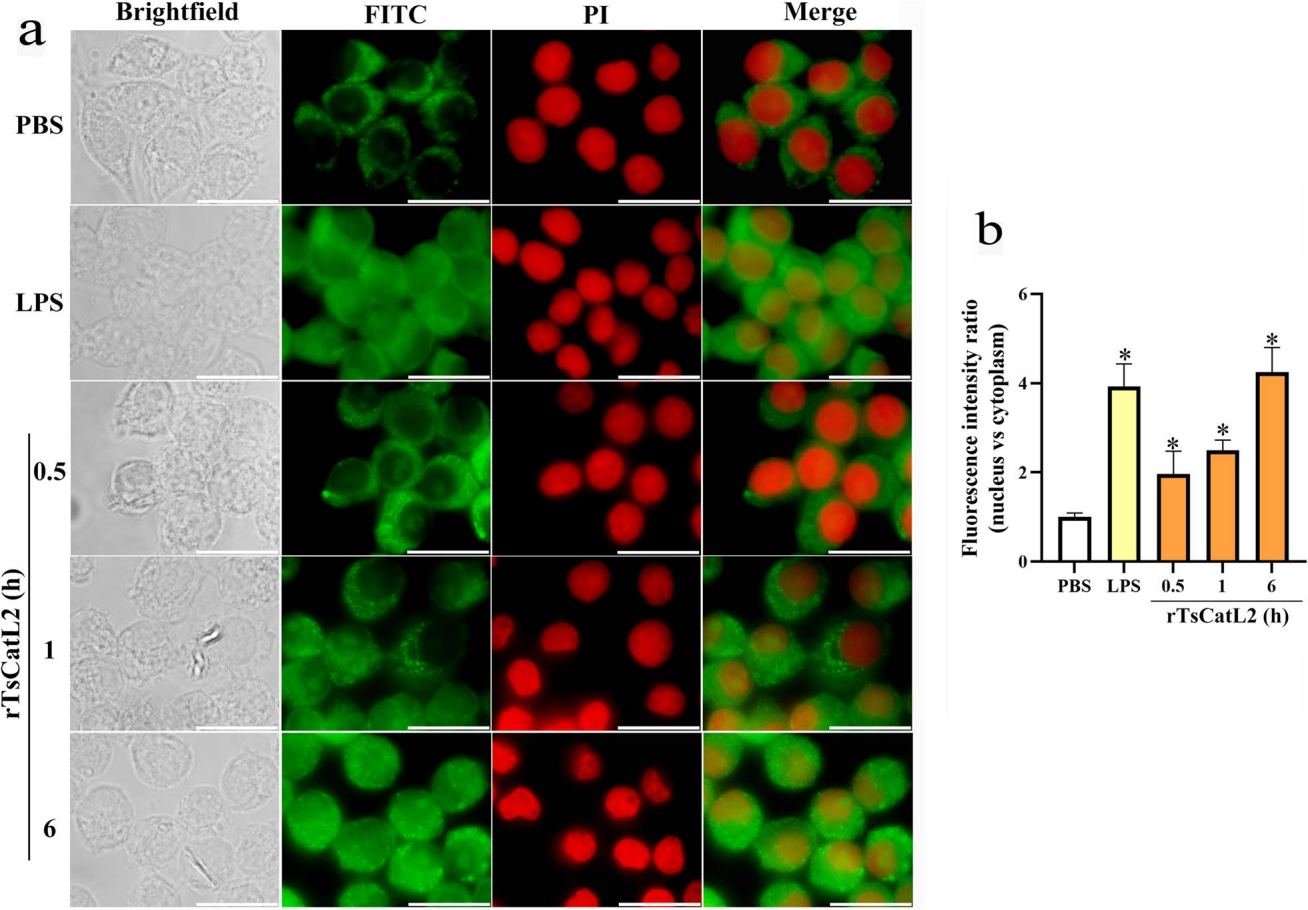
Effect of different incubation times with rTsCatL2 on nuclear translocation of NF-κB p65 protein in RAW264.7 macrophages. RAW264.7 macrophages were stimulated with rTsCatL2 for 0.5 to 6 h. Immunofluorescence showed that NF-κB p65 protein gradually entered the nucleus with increasing stimulation time. Scale bar: 20 μm. The asterisk indicates a statistically significant difference at *P < 0.05 compared with the PBS group. FITC, Fluorescein isothiocyanate; LPS, lipopolysaccharide; NF-κB, nuclear factor kappa-light-chain-enhancer of activated B cells; PBS, phosphate-buffered saline; PI, propidium iodide; RAW264.7, murine macrophage cell line; rTsCatL2, recombinant *T. spiralis* cathepsin L domains

**Fig. 10:**
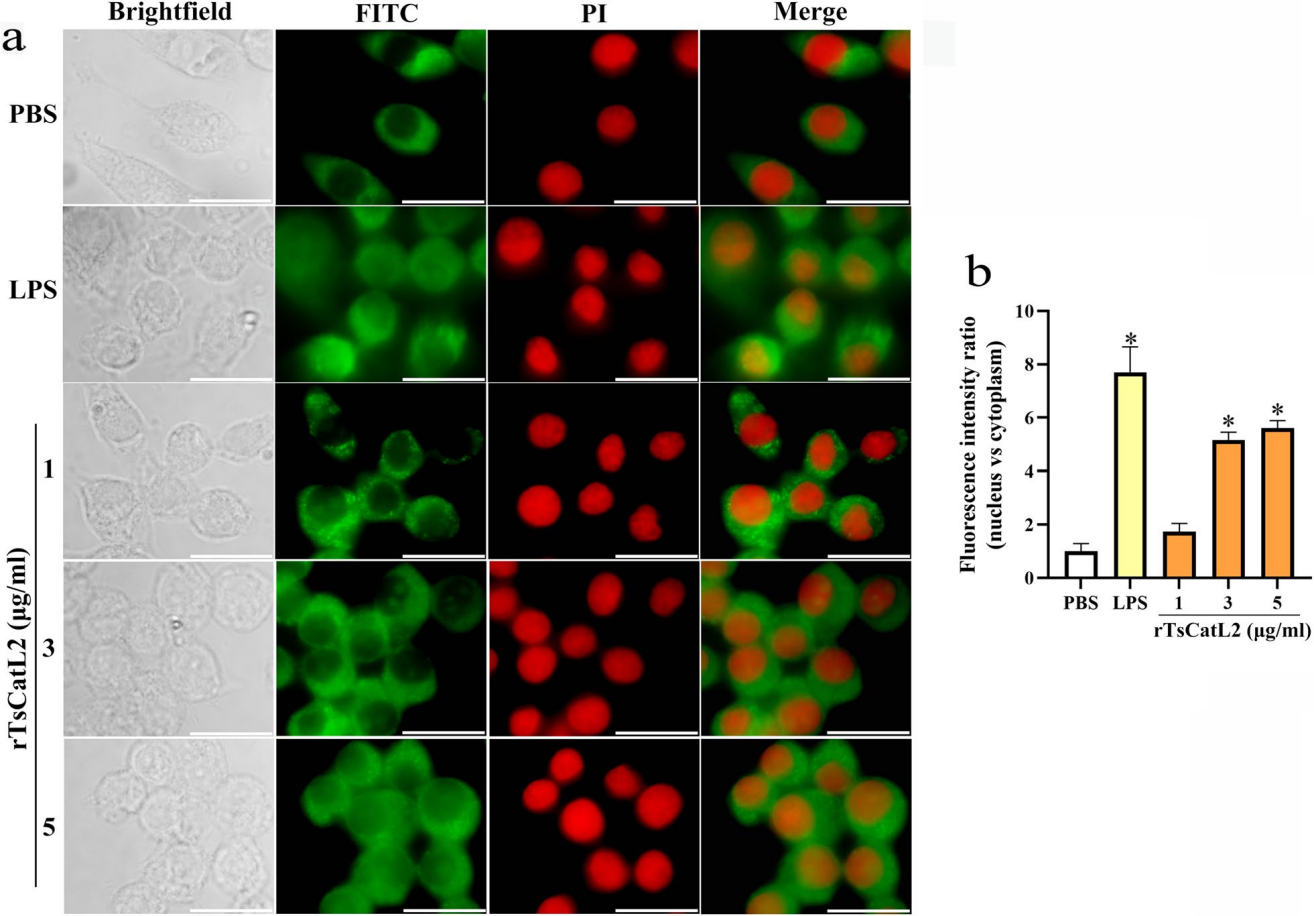
Effect of different concentrations of rTsCatL2 on nuclear translocation of NF-κB p65 protein in RAW264.7 macrophages. Different concentrations of rTsCatL2 (range: 1-5 μg/ml) were used to stimulate RAW264.7 macrophages. Immunofluorescence showed that the nuclear translocation of NF-κB p65 protein increased proportionally with the concentration of rTsCatL2. Scale bar: 20 μm. The asterisk indicates a statistically significant difference at *P < 0.05 compared with the PBS group. FITC, Fluorescein isothiocyanate; LPS, lipopolysaccharide; NF-κB, nuclear factor kappa-light-chain-enhancer of activated B cells; PBS, phosphate-buffered saline; PI, propidium iodide; RAW264.7, murine macrophage cell line; rTsCatL2, recombinant *T. spiralis* cathepsin L domains

The immunofluorescence results demonstrated a clear green fluorescence in the nuclei of all RAW264.7 macrophages with a prolonged incubation time with rTsCatL2 (ANOVA; *F* = 31.733; *P* < 0.05) (Fig. 9). Also, the nuclear translocation of NF-κB p65 protein increased proportionally with the concentration of rTsCatL2 (*r* = 0.907; *P* < 0.01). At rTsCatL2 concentrations of 3 and 5 μg/ml, the nuclear translocation of NF-κB p65 was significantly enhanced (ANOVA; *F* = 94.478, *P* < 0.001) (Fig. 10). In contrast, the PBS group showed no green fluorescence in the nucleus, while the LPS-stimulated positive control group exhibited significant green fluorescence in the nucleus (*P* < 0.001) (Fig. 10). These findings confirm that rTsCatL2 promotes the nuclear translocation of NF-κB p65 protein in RAW264.7 macrophages and that this effect becomes more pronounced with increasing concentrations of rTsCatL2 protein.

### JSH-23 inhibits rTsCatL2-mediated CD86-labeled macrophages proliferation

**Fig. 11:**
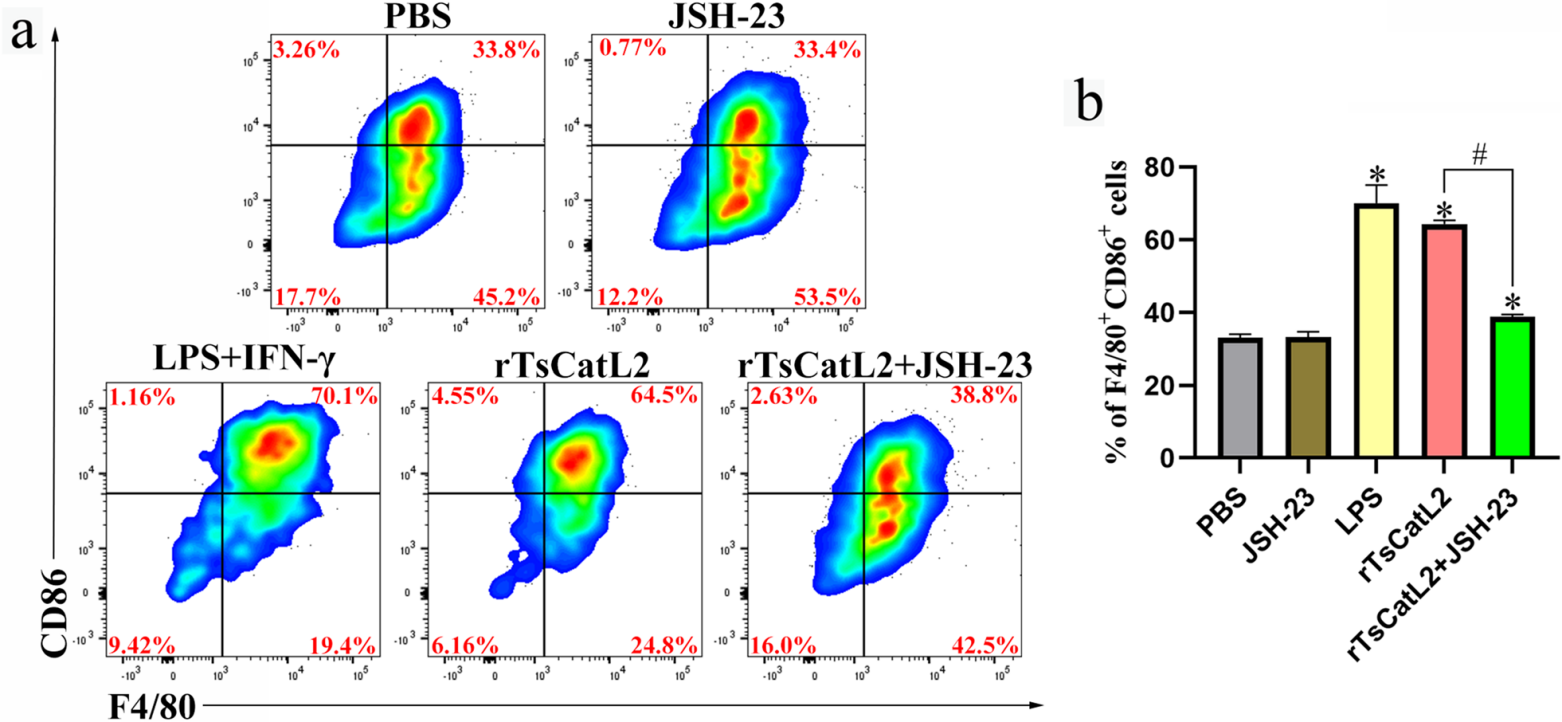
JSH-23 abolishes rTsCatL2-mediated changes of the surface molecules on RAW264.7 macrophages. **a** FlowJo software version 10.8.1 was used to analyze the macrophage population in the different experimental groups. **b** Percentage of M1-type macrophages in the different experimental groups. The asterisk indicates a statistically significant difference at *P < 0.05 compared with the PBS group. The hash sign indicates a statistically significant difference at #*P* < 0.001 between the two groups indicated. IFN, Interferon; JSH-23, inhibitor of NF-κB; LPS, lipopolysaccharide; PBS, phosphate-buffered saline; RAW264.7, murine macrophage cell line; rTsCatL2, recombinant *T. spiralis* cathepsin L domains

The flow cytometry results showed that the proportion of CD86-labeled macrophages increased in both the LPS and rTsCatL2 groups (ANOVA; *F* = 163.086; *P* < 0.001) (Fig. 11). However, the proportion of CD86-labeled macrophages was significantly lower in the rTsCatL2 + JSH-23-treated group compared to the rTsCatL2 group (*P* < 0.001) (Fig. 11). These results suggest that JSH-23 effectively inhibits the rTsCatL2-mediated polarization of M1-type macrophages.

### JSH-23 inhibits rTsCatL2-mediated macrophage pro-inflammatory factor transcription and secretion

**Fig. 12:**
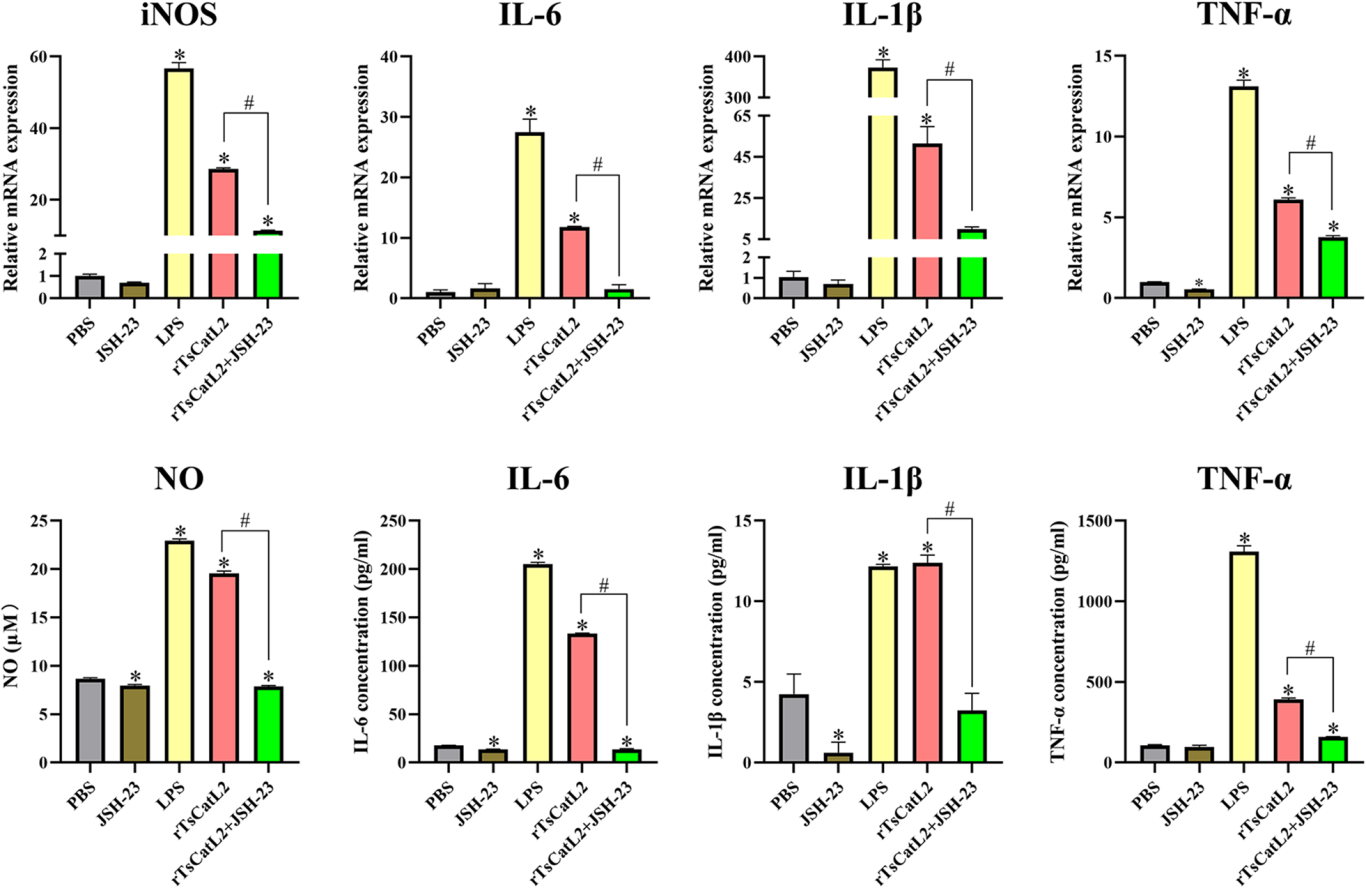
JSH-23 abrogates rTsCatL2-mediated transcription and expression of pro-inflammatory factors in RAW264.7 cells. The transcriptional levels of iNOS, IL-6, IL-1β and TNF-α in macrophages were detected by qPCR. The effects of different treatment groups on the secretion levels of NO, IL-6, IL-1β and TNF-α in macrophages were determined by the Griess and ELISA methods. The asterisk indicates a statistically significant difference at *P < 0.05 compared with the PBS group. The hash sign indicates a statistically significant difference at #*P* < 0.001 between the two groups indicated. JSH-23, inhibitor of NF-κB; iNOS, inducible nitric oxide synthase; INF, interferon; IL, interleukin; LPS, lipopolysaccharide; NO, nitric oxide; mRNA, messenger RNA; PBS, phosphate-buffered saline; RAW264.7, murine macrophage cell line; rTsCatL2, recombinant *T. spiralis* cathepsin L domains; TNF, tumor necrosis factor

The qPCR results indicated significantly higher transcript levels of pro-inflammatory factors, such as iNOS, IL-6, IL-1β and TNF-α, in both the LPS and rTsCatL2 groups compared to the PBS group (ANOVA; *F*_iNOS_ = 3146.192, *F*_IL-6_ = 294.607, *F*_IL-1β_ = 931.27, *F*_TNF-α_ = 2442.571; *P* < 0.001) (Fig. 12). However, in the rTsCatL2 + JSH-23 group, the transcript levels of all the above pro-inflammatory factors were lower than those in the rTsCatL2 group (*P* < 0.01). Similarly, the secretion of pro-inflammatory factors NO, IL-6, IL-1β and TNF-α were significantly increased in both the LPS and rTsCatL2 groups compared to the PBS group, as measured by the Griess and ELISA methods (ANOVA; *F*_NO_ = 6662.9, *F*_IL-6_ = 28,578.646, *F*_IL-1β_ = 139.309, *F*_TNF-α_ = 2610.169; *P* < 0.001). In contrast, the rTsCatL2 + JSH-23 group secreted significantly lower levels of pro-inflammatory factor proteins compared to the rTsCatL2 group alone (*P* < 0.01) (Fig. 12). These results indicate that JSH-23 effectively inhibits rTsCatL2-mediated pro-inflammatory cytokine transcription and secretion in macrophages.

### JSH-23 inhibits rTsCatL2-mediated protein expressions in M1-type macrophages

**Fig. 13:**
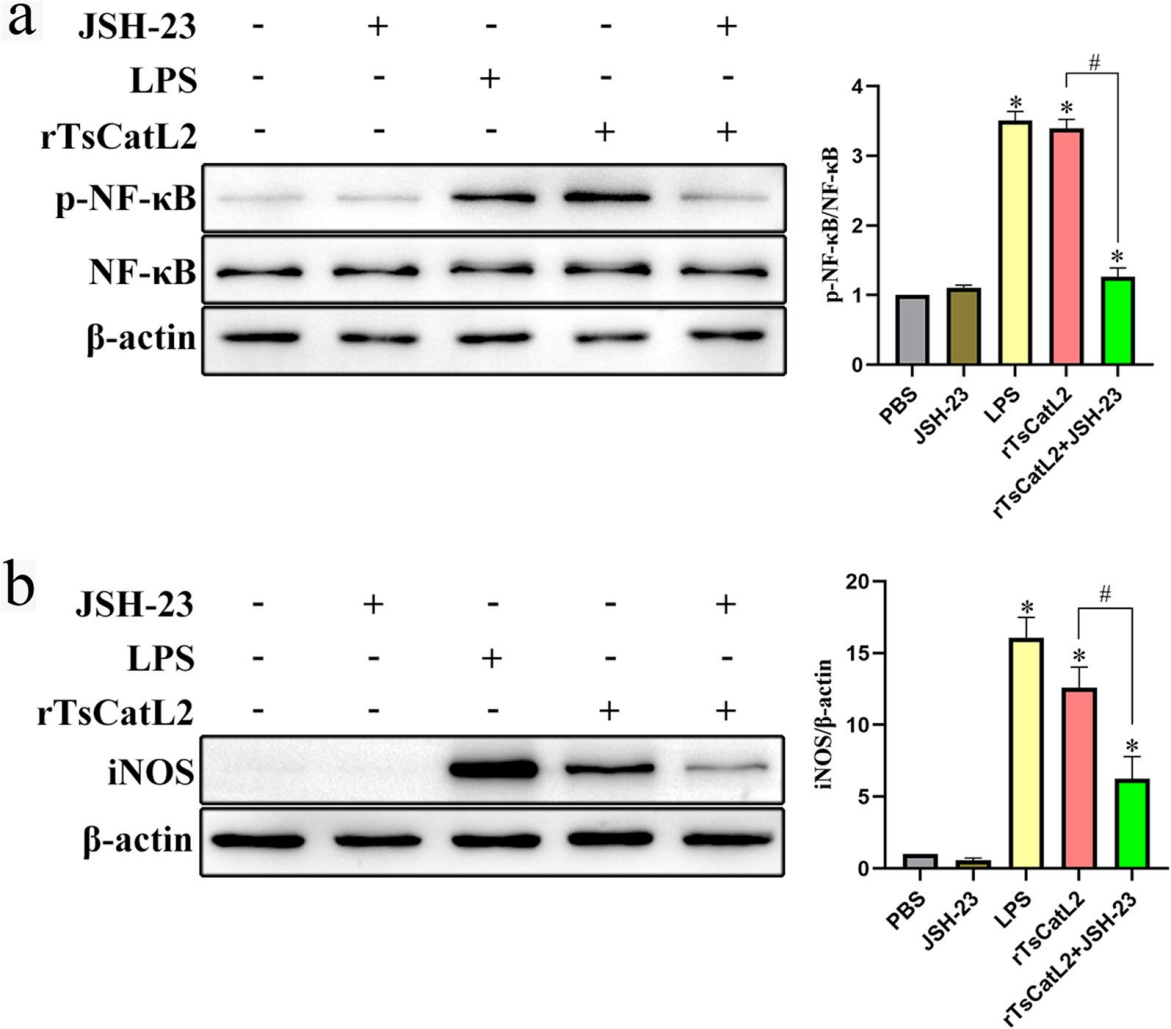
JSH-23 inhibits rTsCatL2-mediated expression of p-NF-κB and iNOS in RAW264.7 macrophages. **a** Effect of different treatments on the NF-κB signaling pathway in macrophages. **b** Effect of different treatments on the expression of iNOS protein in macrophages. The asterisk indicates a statistically significant difference at *P < 0.05 compared with the PBS group. The hash sign indicates a statistically significant difference at #*P* < 0.001 between the two groups indicated. JSH-23, inhibitor of NF-κB; iNOS, inducible nitric oxide synthase; LPS, lipopolysaccharide; NF-κB, nuclear factor kappa-light-chain-enhancer of activated B cells; p, phosphorylated; PBS, phosphate-buffered saline; RAW264.7, murine macrophage cell line; rTsCatL2, recombinant *T. spiralis* cathepsin L domains

According to western blot analysis, the rTsCatL2 group had higher levels of phorphorylated NF-κB protein than the PBS group (ANOVA; *F* = 474.572; *P* < 0.001). However, pre-treatment of cells with JSH-23 inhibited the rTsCatL2-mediated phosphorylation of NF-κB protein (*P* < 0.01) (Fig. 13a). Additionally, the expression level of iNOS protein was higher in the rTsCatL2 group than in the PBS group (ANOVA; *F* = 112.143; *P* < 0.001). Nevertheless, pre-treatment of cells with JSH-23 reduced the rTsCatL2-mediated expression of iNOS in RAW264.7 macrophages (*P* < 0.01) (Fig. 13b).

### rTsCatL2 enhances the direct killing effect of M1 macrophages on NBL

**Fig. 14:**
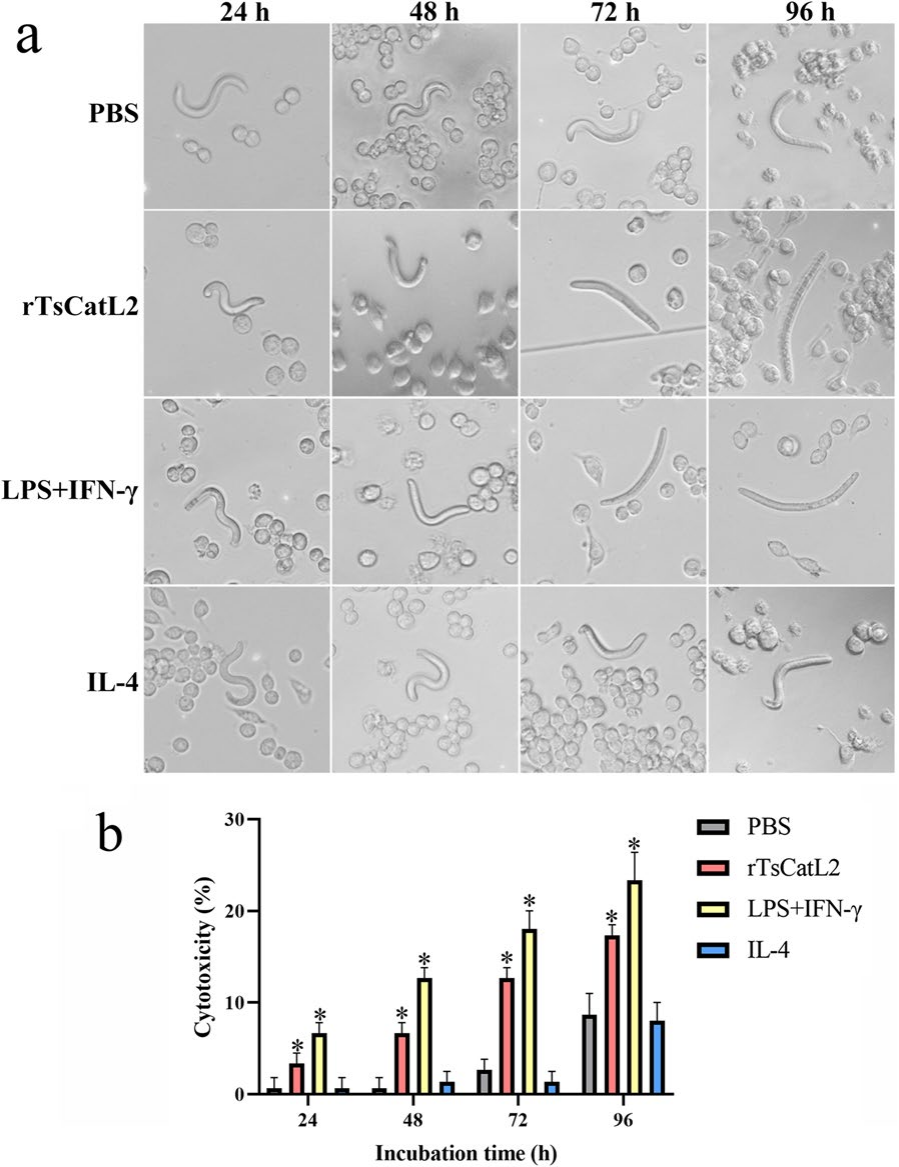
rTsCatL2 enhances the macrophage's direct killing effect on NBL. **a** Microscopic observation of macrophage’s direct killing effect on NBL. **b** Mortality of NBL after incubation with rTsCatL2 for different lengths of time. The asterisk indicates a statistically significant difference at *P < 0.05 compared with the PBS group. Scale bar: 100 μm. IFN, Interferon; IL, interleukin; LPS, Lipopolysaccharide; NBL, newborn larvae; PBS, phosphate-buffered saline; RAW264.7, murine macrophage cell line; rTsCatL2, recombinant *T. spiralis* cathepsin L domains

RAW264.7 cells were stimulated with PBS, IL-4, LPS + IFN-γ or rTsCatL2 for 6 h, following which NBL were added and cultured for 96 h. Microscopic examination did not detected any macrophage adhesion around the NBL. The NBL mortality rate in the rTsCatL2 and LPS + IFN-γ groups was higher than that in the PBS and IL-4 groups (Chi-square test; *χ*^*2*^_24h_ = 8.247, *χ*^*2*^_48h_ = 19.048, *χ*^*2*^_72h_ = 24.642, *χ*^*2*^_96h_ = 12.337; *P* < 0.05). Specifically, the NBL mortality rates in the rTsCatL2 group were 9.33%, 32%, 58% and 79.33% at 24, 48, 72 and 96 h, respectively (Fig. 14). These findings suggest that after rTsCatL2 promoted the polarization of M1-type macrophages, cytokines secreted by M1-type macrophages enhanced the killing effect on NBL.

.

### rTsCatL2 enhances the synergistic killing of macrophages and complement

**Fig. 15:**
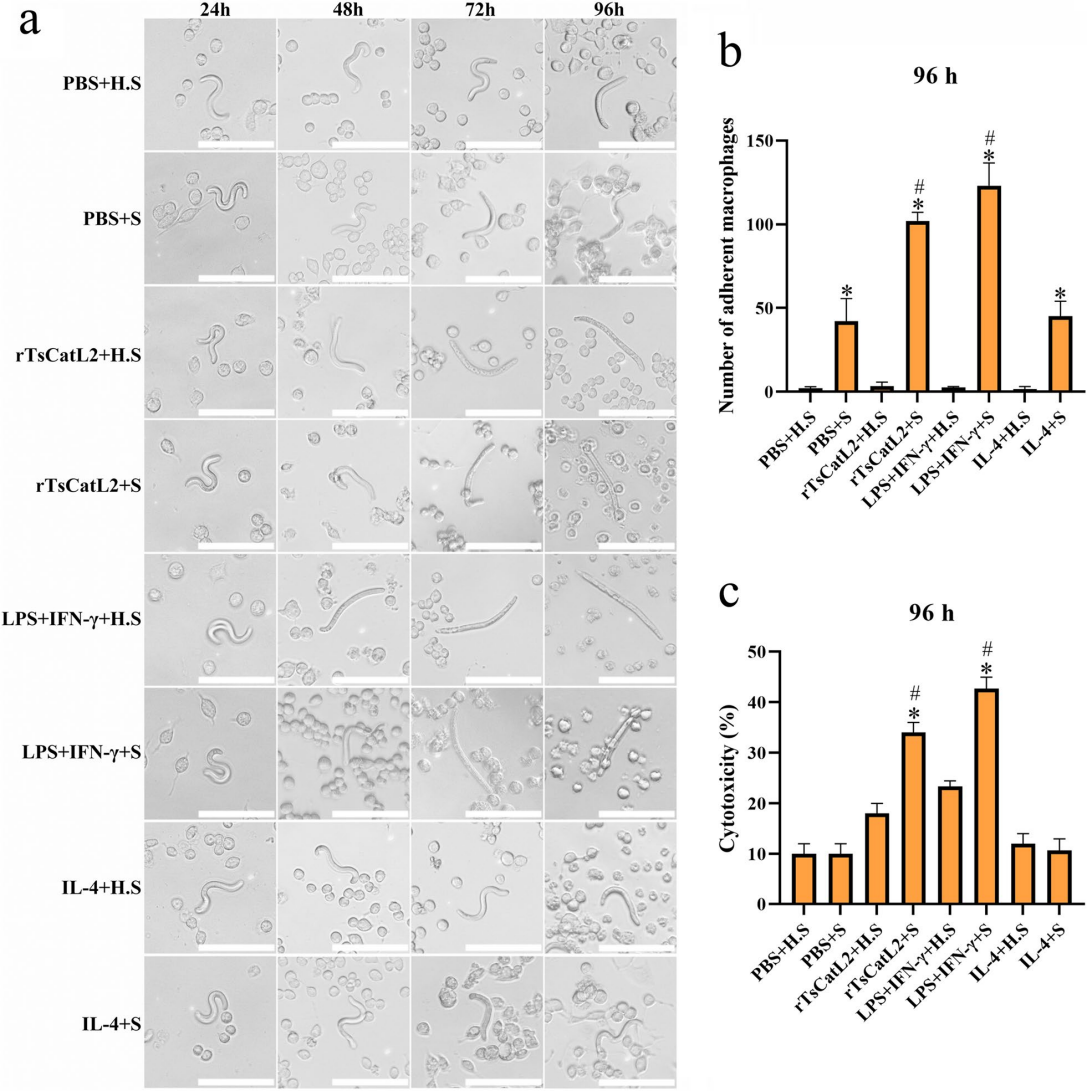
Complement enhances rTsCatL2-mediated M1-type macrophage killing on NBL. **a** Microscopic observation of macrophage adhering to and killing of NBL. **b** Number of macrophages adhering to NBL after 96 h of incubation. **c** Mortality rate of NBL after incubation for 96 h. Scale bar: 100 μm. The asterisk represents a statistically significant difference between the fresh guinea pig serum (S) group and the guinea pig serum with inactivated complement (H.S) group at *P* < 0.05). The hash sign indicates that the indicated effects of the rTsCatL2 + S and LPS + IFN-γ + S groups were significantly higher than those of the PBS + S group and IL-4 + S group at #*P* < 0.05. IFN, Interferon; IL, interleukin; LPS, Lipopolysaccharide; NBL, newborn larvae; PBS, phosphate-buffered saline; RAW264.7, murine macrophage cell line; rTsCatL2, recombinant *T. spiralis* cathepsin L domains

RAW264.7 cells were stimulated with PBS, IL-4, LPS + IFN-γ or rTsCatL2 for 6 h, following which complement and NBL were added and cultured for 96 h. The added complement was divided into fresh guinea pig serum (S) and guinea pig serum with inactivated complement (H.S). The results showed that macrophage adhesion to NBL was higher in the group treated with S than in that treated with H.S (t-test; *t*_PBS_ = 5.026, *t*_rTsCatL2_ = 29.6, *t*_LPS+IFN-γ_ = 15.147, *t*_IL-4_ = 8.222; *P* < 0.05). The number of adherent cells around NBL in the rTsCatL2 + S group and LPS + IFN-γ + S group was significantly higher than that in the PBS + S group and IL-4 + S group (ANOVA; *F* = 41.037; *P* < 0.001) (Fig. 15a b). Additionally, complement enhanced the killing effect of M1-type macrophages on NBL. The NBL mortality rate in the rTsCatL2 + S group was 34%, which was higher than that in the rTsCatL2 + H.S group (18%) (Chi-square test; *χ*^2^ = 6.653; *P* < 0.05); the NBL mortality rate in the LPS + IFN-γ + S group was 42.67%, which was higher than that of the LPS + IFN-γ + H.S group (23.33%) (Chi-square test; *χ*^2^ = 9.046; *P* < 0.05). However, the addition of complement had little effect on macrophage killing in the PBS and IL-4 groups (*P* > 0.05) (Fig. 15c). These results indicate that rTsCatL2 can further enhance macrophage adhesion and the killing effect on NBL through the synergistic effect of complement.

### rTsCatL2 enhances ADCC mediated by macrophages and antibodies

**Fig. 16:**
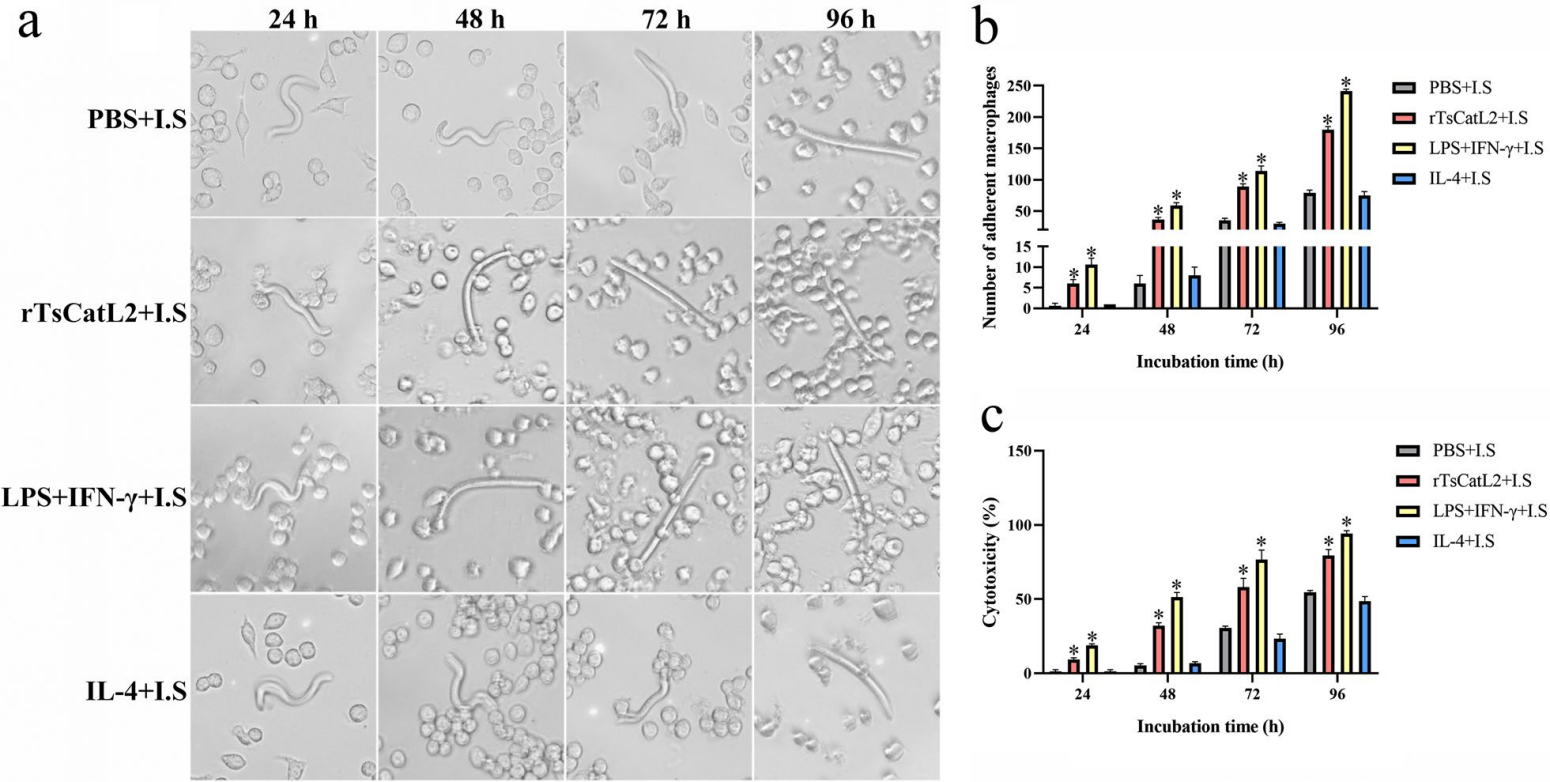
rTsCatL2 enhances the killing effect of macrophages on NBL via ADCC. **a** Addition of rTsCatL2 immune serum (I.S) enhanced rTsCatL2-mediated killing of NBL by M1-type macrophages. **b** The number of macrophages adherent to NBL in various groups. **c** Mortality of NBL in diverse groups. The asterisk indicates a statistically significant difference at *P < 0.05 compared with the PBS group at different time points. Scale bar: 100 μm. ADCC, Antibody-dependent cell-mediated cytotoxicity; IFN, interferon; IL, interleukin; LPS, Lipopolysaccharide; NBL, newborn larvae; PBS, phosphate-buffered saline; RAW264.7, murine macrophage cell line; rTsCatL2, recombinant *T. spiralis* cathepsin L domains

RAW264.7 cells were stimulated with PBS, IL-4, LPS + IFN-γ or rTsCatL2 for 6 h, following which rTsCatL2 immune serum (I.S) and NBL were added and cultured for 96 h. The adhesion numbers of macrophages in the rTsCatL2 + I.S and LPS + IFN-γ + I.S groups were significantly higher than those in the PBS + I.S and IL-4 + I.S groups (ANOVA; *F*_24h_ = 73.303, *F*_48h_ = 186.581, *F*_72h_ = 208.14, *F*_96h_ = 877.672; *P* < 0.001) (Fig. 16a, b). Additionally, the ADCC results showed that the NBL mortality rates of the rTsCatL2 + I.S and the LPS + IFN-γ + I.S groups were significantly higher than those of the PBS + I.S and IL-4 + I.S groups (Chi-square test; *χ*_24h_^2^ = 31.568, *χ*_48h_^2^ = 79.669, *χ*_72h_^2^ = 74.335, *χ*_96h_^2^ = 62.023; *P* < 0.001) (Fig. 16c). At 24, 48, 72 and 96 h of ADCC, the NBL mortality rate of the rTsCatL2 + I.S group was 9.33%, 32%, 58% and 79.33%, respectively, indicating that rTsCatL2 significantly enhanced the ADCC effect of macrophages by promoting macrophage M1 polarization.

## Discussion

The severity of trichinellosis symptoms depends on the quantity of *T. spiralis* larvae ingested and the host's immunological response [41]. Upon host infection, innate and adaptive immune responses are induced, and it has been shown that helminth-related proteins have therapeutic potential in various host pathological and inflammatory diseases [42]. Cathepsin L, a lysosomal cysteine proteolytic enzyme, significantly impacts the immunological response of the host and host-parasite interaction [43, 44]. In the present study, *T. spiralis* cathepsin L was successfully induced using the *E. coli* prokaryotic expression system, and rTsCatL2 was identified as *T. spiralis* cathepsin L (KRY31298.1) through LC–MS/MS.

Macrophages, which are critical congenital immune cells, are vital for killing invading pathogens by phagocytizing foreign particles and participating in immune responses, thus maintaining homeostasis in the internal environment [45]. Studying the interaction between rTsCatL2 and macrophages is essential for further understanding the mechanism of trichinellosis. The results of the CCK-8 assay indicated that rTsCatL2 promoted macrophage proliferation at a specific concentration and that when the concentration of rTsCatL2 was 5 μg/ml for 48 h, the viability of macrophages was not reduced. CCK-8 is a redox indicator that dynamically counts living cells and can identify medication toxicity and cell proliferation [46]. Immunofluorescence assays confirmed the binding of rTsCatL2 to macrophages, indicating its activation effect on macrophages.

Macrophages can undergo polarization into M1 macrophages when stimulated by cytokines, pathogens or LPS. The iNOS enzyme utilizes NADPH and oxygen to produce NO, a critical effector secreted by macrophages and contributing to the inflammatory response [47]. Additionally, M1-type macrophages secret pro-inflammatory cytokines, including IL-6, IL-1β and TNF-α, which can attract neutrophils, eosinophils and T cells, modulating the immune response [48]. The findings of this investigation indicated that rTsCatL2 significantly promoted the transcription and secretion of iNOS, IL-6, IL-1β and TNF-α, and increased the number of CD86-labeled macrophages. Our findings support the role of rTsCatL2 in promoting M1 polarization of RAW264.7 macrophages. Research has demonstrated that intestine, mesenteric lymph nodes and spleen macrophages are M1-type activated after *T. spiralis*-infected mice at 1 and 5 days post-infection [12]. Human THP-1 macrophages secrete pro-inflammatory cytokines such as IL-1β and TNF-α when co-cultured with mature *T. spiralis* worms or NBL in vitro [49]. rTsCatL2 has the highest mRNA transcript level in the *T. spiralis* infective larval stage in the intestine [20]. In the initial stages of trichinellosis, macrophages tend to polarize towards the M1-type, promoting an inflammatory response.

The NF-κB pathway is one of the most studied pathways that promote M1-type polarization of macrophages, and phosphorylation levels of NF-κB and IκB-α indicate activation of the pathway [50]. Activation of the NF-κB pathway also leads to translocation of NF-κB p65 protein from the cytoplasm to the nucleus, where it binds to the κB elements of target genes and induces the transcription of pro-inflammatory cytokines, such as IL-6, IL-1β and TNF-α [51, 52]. Interestingly, our study demonstrated that rTsCatL2 increased the phosphorylation levels of NF-κB and IκB-α and activated the NF-κB signaling pathway. rTsCatL2 effectively promoted the nuclear translocation of NF-κB p65 protein in RAW264.7 macrophages, with this effect becoming more pronounced with increasing duration of stimulation time and increasing concentration. The study demonstrated that rTsCatL2 promotes the polarization of RAW264.7 macrophages towards the M1 phenotype by enhancing NF-κB signaling pathway activation.

In the present study, RAW 264.7 macrophages were pretreated with 30 μM JSH-23, which inhibited NF-κB protein phosphorylation and iNOS protein production. Furthermore, JSH-23 downregulated the transcriptional activity of iNOS, IL-6, IL-1β and TNF-α, decreasing their protein secretion levels. Flow cytometry analysis further confirmed a significant reduction in the proportion of M1 macrophages. Concentrations of JSH-23 ranging from 3 to 30 μM reduced NF-κB p65 protein levels in LPS-stimulated RAW 264.7 cells [38, 53]. Moreover, JSH-23 effectively inhibited the secretion of pro-inflammatory cytokines IL-6, IL-1β, COX-2 and TNF-α induced by LPS [38, 53]. These findings indicate that JSH-23 can effectively inhibit rTsCatL2-induced M1-type macrophage polarization by blocking the NF-κB signaling pathway.

In addition to direct pathogen elimination, macrophages can kill NBL through complement or ADCC mechanisms. The death rate of NBL significantly increased in the rTsCatL2 stimulation group, indicating that M1-type macrophages may play a killing role by secreting cytokines such as TNF-α and IFN-γ, reactive oxygen species or NO. Complement serum significantly enhanced macrophages’ adhesion and killing ability in the rTsCatL2 stimulation group, possibly due to complement opsonophagocytosis, which improves macrophage adhesion and pathogen phagocytosis. Additionally, rTsCatL2 promoted the ADCC effect of macrophages by inducing polarization toward M1-type macrophages. M1-type macrophages demonstrate high expression of cytokines, including TNF-α, IL-1β, IL-6 and reactive oxygen and nitrogen intermediates. These factors significantly enhance the phagocytic function of macrophages, enabling the effective elimination of infected parasites [54]. Additionally, M1 macrophages secrete abundant NO, contributing to the eradication of parasites like *Plasmodium yoelii*, *Leishmania*, *Schistosoma mansoni* and *Toxoplasma gondii* [54–57]. NO has demonstrated efficacy in combating *T. spiralis* infection by reducing worm load [58, 59]. Consequently, rTsCatL2 likely regulates the host immune response, augmenting macrophage-mediated killing by promoting M1 polarization. Further in vivo experiments in mice can shed light on the effects of rTsCatL2 on the host immune response, significantly advancing our understanding of trichinellosis immunopathology and potential treatment strategies. Our study has a number of limitations, which can be followed up in future studies aimed at exploring whether JSH-23 can block rTsCaL2 to enhance macrophage NBL killing. Animal experiments can also be used to evaluate the immunomodulation of mice and the killing effect on *T. spralis* after rTsCaL2 immunization. Furthermore, future research could investigate other receptor proteins linked to macrophages by rTsCatL2 and modifications to critical molecules in the NF-κB pathway.

In conclusion, co-incubation of rTsCatL2 with RAW264.7 macrophages induced M1-type macrophage polarization by activating the NF-κB signaling pathway. As a result, macrophages exhibited increased efficacy in killing NBL. This study provides a theoretical basis for the prevention and treatment of trichinellosis.

## Data Availability

The data supporting the conclusions of this article have been included within the article.

## Abbreviations

AAM or M2: Alternatively activated macrophages (M2 phenotype)
ADCC: Antibody-dependent cell-mediated cytotoxicity
Arg-1: Arginase-1
CAM or M1: Classically activated macrophages (M1 phenotype)
CCK-8: Cell Counting Kit-8
H.S: Inactivated complement sera
IκB-α: Nuclear factor-κB inhibitor alpha
IL: Interleukin
iNOS: Inducible nitric oxide synthase
I.S: RTsCatL2 immune serum
LPS: Lipopolysaccharide
NBL: Newborn larvae
NF-κB: Nuclear factor kappa-light-chain-enhancer of activated B cells
NC: Nitrocellulose filter
NO: Nitric oxide
PI: Propidium iodide
OD: Optical density
S: Normal serum
rTsCatL2: Recombinant *T. spiralis* cathepsin L domains
TsCatL: *Trichinella spiralis* cathepsin L
TNF-α: Tumor necrosis factor alpha
